# Peripheral Coordination-Dependent Descriptor for Selective Interactions between Near-Frontier Molecular Orbitals and Single-Atom Catalysts

**DOI:** 10.1021/prechem.3c00015

**Published:** 2023-04-13

**Authors:** Bingqing Ge, Fenfei Wei, Qiang Wan, Hongwei Zhang, Pei Yuan, Sen Lin

**Affiliations:** † State Key Laboratory of Photocatalysis on Energy and Environment, College of Chemistry, 12423 Fuzhou University, Fuzhou 350002, China; ‡ National Engineering Research Center of Chemical Fertilizer Catalyst, College of Chemical Engineering, 12423 Fuzhou University, Fuzhou 350002, China

**Keywords:** selectivity, adsorption, coordination environment, DFT, descriptor

## Abstract

Selective adsorption of α,β-unsaturated aldehydes (α,β-UALs) is a prerequisite for the hydrogenation of α,β-UALs to high-value unsaturated alcohols, but a quantitative description of the interactions between the C=C/C=O bond of α,β-UALs and the catalysts is still lacking. Herein, based on comprehensive density functional theory calculations, we developed a descriptor that combines the near-frontier molecular orbitals of the C=C/C=O bonds of α,β-UALs with the fundamental physical properties of single-atom catalysts (SACs) and considers the inner/outer coordination environment. All of the parameters used in this descriptor are easily accessible and interpretable, enabling an efficient assessment of the selectivity of SACs for the C=C/C=O bonds of α,β-UALs.

## Graphical abstract

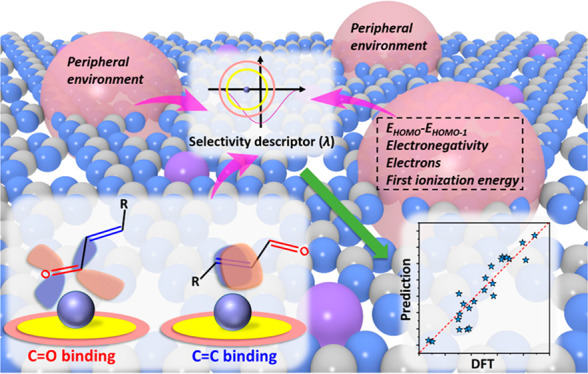

## 1 Introduction

Selective hydrogenation of C=O bonds of α,β-unsaturated aldehydes (α,β-UALs) to unsaturated alcohols (α,β-UOLs) is a challenging class of reactions, yielding valuable fine chemicals, such as pharmaceuticals, fragrances, and flavors.^1–3^ On conventional metal catalysts, such as Pd(111) and Pt(111), the hydrogenation of the C=O bonds of α,β-UALs is thermodynamically less favorable than that of the C=C bond, which can lead to the undesired saturated aldehyde.^4–7^ Hence, new catalysts are urgently needed to achieve the target products.

An understanding of adsorbate–surface interactions is essential for the design of catalysts with high selectivity,^8,9^ but this understanding is often elusive. For example, experimental studies on both single-crystal surfaces and powdered materials have clearly shown that the C=C bond of acrolein (C_3_H_4_O) is preferentially hydrogenated over the clean Pd(111) surface while theoretical calculations failed to explain these observations and instead have suggested the hydrogenation of the C=O bond on this metal surface.^10,11^ In a recent theoretical study on α,β-UAL hydrogenation over Pt(111), Liu et al. for the first time attributed the experimentally observed selectivity to a significantly larger broadening of the highest occupied molecular orbital – 1 (HOMO – 1) of the C=C bond than that of the HOMO of the C=O bond.^12^ Spivey and Holewinski demonstrated by theory that the selective adsorption behavior of α,β-UAL on single-atom alloys could be attributed to the interaction between the HOMO in the C=O bond or the HOMO – 1 in the C=C bond with the sharp single-atom-like electronic d-states located on the alloy composition.^13^ Although these studies have qualitatively argued that the selective adsorption of α,β-UALs depends mainly on the molecular orbital (MO) interactions between the C=C/C=O bonds of α,β-UALs and the catalyst surface, a quantitative descriptions is still lacking.

Single-atom catalysts (SACs)^14^ have been widely used for selective hydrogenation reactions.^15,16^ Thanks to its well-defined single-site geometry, SAC can avoid the adsorption of multiple unsaturated bonds at one time, and therefore, it is an ideal model for the study of adsorbate–surface interaction. In particular, the adsorbate–surface interaction can be tuned by modifying the coordination environment (CE) of SACs through various chemical means.^17–20^ Encouragingly, this approach offers the potential to achieve selective adsorption of α,β-UALs at single-atom sites with the C=O bond rather than the C=C bond.

In this work, based on comprehensive density functional theory (DFT) calculations, we developed a simple descriptor by combining the MOs of the C=C/C=O bonds of α,β-UALs with the basic physical properties of the CE and the single atom in SACs. Importantly, all of the parameters in this descriptor are easily accessible and understood, thus enabling a rapid prediction of the selectivity of SACs for the C=C/C=O bonds of α,β-UALs.

## 2 DFT Calculation Details

All spin-polarized DFT calculations were performed using the Vienna ab initio simulation package (VASP) based on plane-wave basis sets.^21,22^ Electron–ion interactions were described using projector augmented wave (PAW) potentials^23^ with an energy cutoff of 400 eV. The generalized gradient approximation (GGA) with the Perdew–Burke–Ernzerhof (PBE) functional was adopted for structural optimization.^24^ The van der Waals correction was included using the DFT-D3 method of Grimme.^25^

A 2 × 2 supercell of the C_2_N monolayer and *g*-C_3_N_4_ monolayer and a 6 × 6 supercell of defective graphene and *h*-BN monolayer were used to support the single metal atoms. The distance between the monolayer and its neighboring images exceeds 20 Å, which is sufficient to avoid interactions between them. The Brillouin zones were simulated by using a Monkhorst–Pack (MP) grid of 3 × 3 × 1. All minima geometries were optimized until the total energy was converged to 10^–4^ eV and the forces acting on each atom were converged below 0.05 eV Å^–1^.

1 $$E_{\mathrm{ads}}=E_{\mathrm{total}}-E_{\mathrm{substrate}}-E_{\mathrm{adsorbate}}$$

2 $$\Delta E_{\mathrm{ads}}=E_{\mathrm{ald}\mathrm{‐mode}}-E_{\mathrm{ene}\mathrm{‐mode}}$$

where *E*_ads_ is the adsorption energy of the molecule, *E*_total_ is the total energy of the system, *E*_substrate_ is the energy of the catalyst, and *E*_adsorbate_ is the energy of the molecule. *E*_*ald*‑mode_ and *E*_*ene*‑mode_ are the adsorption energies of α,β-UALs following the *ald*-mode and *ene*-mode, respectively.

The CE in this work is considered to be composed of atomic components within a truncated radius from the central atom. Previous artificial neural network (ANN) studies have suggested that the energy of an atom was influenced by atoms within a range of about 6 Å.^26^ Therefore, here a cutoff radius (*r*_*ij*_) of 6 Å was used in M_1_@C_2_N and M_1_@*g*-C_3_N_4_, while 5.5 Å was used for M_1_@divacancy-graphene (DG) and M_1_@hexagonal boron nitride (*h*-BN) to ensure that the absolute values of the descriptors were in a similar range as the number of adjacent atoms decreases due to the presence of holes in M_1_@C_2_N and M_1_@*g*-C_3_N_4_.

## 3 Results and Discussion

### 3.1 Adsorption Models of α,β-UALs on SACs

We adopted four typical α,β-UALs, including C_3_H_4_O, C_4_H_6_O, 2-methyl-2-butenal (C_5_H_8_O), and 2-pentenal (Figure 1a) adsorbed on M_1_@C_2_N,^27^ M_1_@*g*-C_3_N_4_,^28^ M_1_@DG,^29^ and M_1_@*h*-BN^30^ SACs (Figure 1b) yielding more than 800 models. See the details in the Supporting Information. On each SAC, α,β-UAL has two adsorption modes, denoted as *ald*-mode and *ene*-mode (Figure 1c), which lead to Route 1 and Route 2 (Figure 1d), respectively. As shown in Figures S1–S4, the adsorption of C_3_H_4_O could follow only one mode at a time on each SAC.

**Figure 1:**
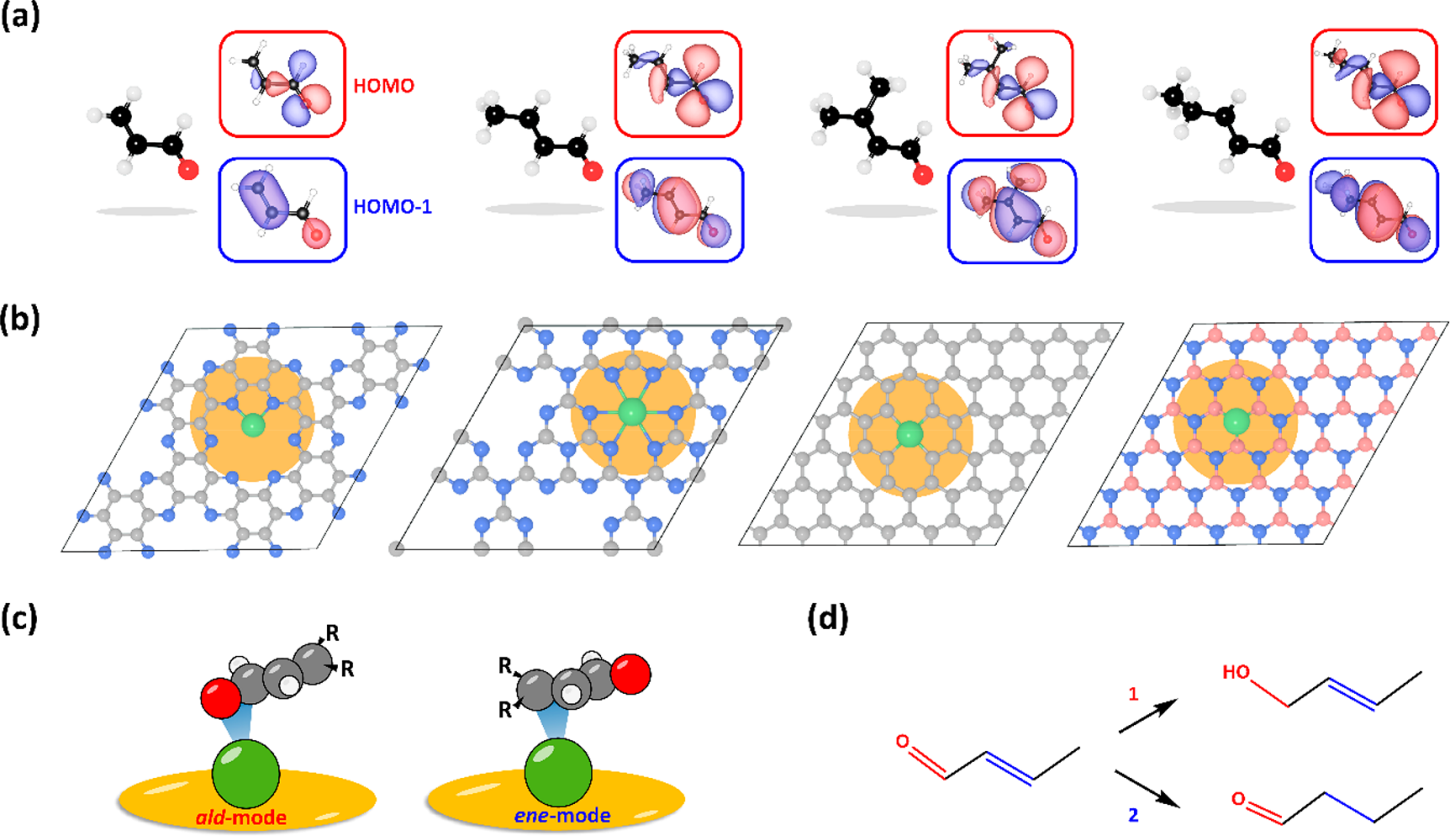
(a) α,β-UALs, including C_3_H_4_O, C_4_H_6_O, C_5_H_8_O, and 2-pentenal, with their HOMO and HOMO – 1 orbitals. White: H; black: C; red: O. (b) SAC models of M_1_@C_2_N, M_1_@*g*-C_3_N_4_, M_1_@DG, and M_1_@*h*-BN. Pink: B; gray: C; blue: N; green: M_1_. (c) Schemes of α,β-UALs adsorbed on SACs with *ald*-mode and *ene*-mode and (d) reaction routes for the hydrogenation of C=O/C=C bonds of α,β-UALs.

### 3.2 The Construction of the Descriptor

To obtain atomistic insights into the interaction between the C=O/C=C bond and the M_1_ site, we performed electronic structure analysis. Figure 1a clearly shows that the near-frontier MOs (HOMO and HOMO – 1) of α,β-UALs derive contributions from C=O and C=C bonds, respectively, whose interactions with the d-orbitals of M_1_ yield different adsorption energies (*E*_ads_) (Tables S1–S4). The HOMO/HOMO – 1 energies for the molecules are listed in Table S5, and the definition of *E*_ads_ and the difference of *E*_ads_ between the *ald*-mode and *ene*-mode (Δ*E*_ads_) are shown in the Supporting Information. Next, we analyzed the partial density of states (PDOSs) of C_3_H_4_O adsorbed on M_1_@C_2_N (M = Ti, Pt, and Mn) to understand the interactions between SACs and α,β-UALs. We found that the higher d-band of Ti_1_ has more overlap with the HOMO of C_3_H_4_O and the molecule preferred to adsorb in the *ald*-mode (Figure 2a), yielding a Δ*E*_ads_ of −0.65 eV. In contrast, the lower d-band of Pt_1_ has more significant coupling with the HOMO – 1 of C_3_H_4_O, making the molecule adsorb in the *ene*-mode (Figure 2b), where Δ*E*_ads_ is 1.17 eV. For C_3_H_4_O adsorbed on Mn_1_@C_2_N, both the HOMO and HOMO – 1 of the molecule have a large overlap with the d-band of Mn_1_ in a moderate energy region (Figure 2c), such that Δ*E*_ads_ is close to zero. The PDOSs for other systems are displayed in Figures S5–S20.

**Figure 2:**
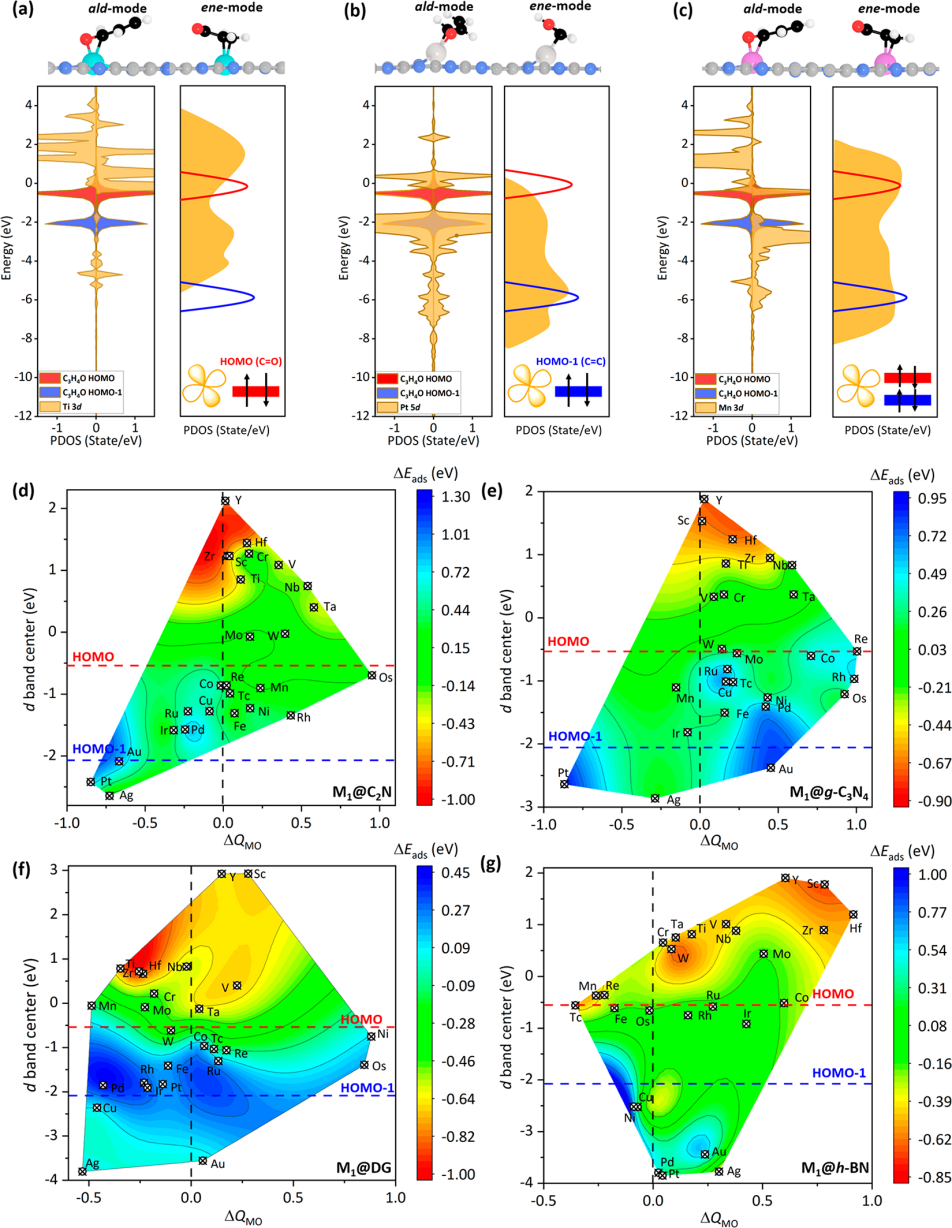
Optimized adsorption structures of C_3_H_4_O in *ald*-mode and *ene*-mode, and the HOMO (red) and HOMO – 1 (blue) of C_3_H_4_O and the PDOSs for d-states of central metal atoms for (a) Ti_1_@C_2_N, (b) Pt_1_@C_2_N, and (c) Mn_1_@C_2_N. Cyan: Ti; silver: Pt; fuchsia: Mn; gray: C; blue: N. The 2D contour diagrams of Δ*E*_ads_ of C_3_H_4_O as a function of the d-band center of M_1_ with the difference between the d-band and MO overlap (Δ*Q*_MO_) on (d) M_1_@C_2_N, (e) M_1_@*g*-C_3_N_4_, (f) M_1_@DG, and (g) M_1_@*h*-BN.

To better elucidate the correlation of Δ*E*_ads_ with the d-band of M_1_ and the MO of α,β-UALs, we plotted two-dimensional (2D) contour plots of Δ*E*_ads_ of C_3_H_4_O adsorbed on the four SACs as a function of the d-band center and the difference between the d-band and MO overlap (Δ*Q*_MO_; see eq 3) (Figures 2d–2g). As shown in the results, SACs with early transition metals tended to adsorb the C=O bond of α,β-UALs (warm colors), whereas the SACs with post-transition metals bound more readily to the C=C bond of α,β-UALs (cool colors). In general, the *ald*-mode is more favorable than the *ene*-mode when the d-band center of M_1_ is located above the HOMO and the Δ*Q*_MO_ value is larger than zero (the upper-right region). This finding certainly enhances our understanding of the origin of the electronic structure of the selective adsorption of α,β-UALs. The 2D plots for the other three molecules adsorbed on the four SACs are shown in Figures S21–S23 and present similar results.

3 $$\Delta Q_{\mathrm{MO}}=Q_{\mathrm{HOMO}}-Q_{\mathrm{HOMO}-1}$$

4 $$Q_{\mathrm{HOMO}}=\int_{-\infty }^{0}\left[\min\left(n_{d,\mathrm{up}}\left(\varepsilon \right),n_{\mathrm{HOMO},\mathrm{up}}\left(\varepsilon \right)\right)-\max\left(n_{d,\mathrm{down}}\left(\varepsilon \right),n_{\mathrm{HOMO},\mathrm{down}}\left(\varepsilon \right)\right)\right]d\varepsilon$$

5 $$Q_{\mathrm{HOMO}-1}=\int_{-\infty }^{0}\left[\min\left(n_{d,\mathrm{up}}\left(\varepsilon \right),n_{\mathrm{HOMO}-1,\mathrm{up}}\left(\varepsilon \right)\right)-\max\left(n_{d,\mathrm{down}}\left(\varepsilon \right),n_{\mathrm{HOMO}-1,\mathrm{down}}\left(\varepsilon \right)\right)\right]d\varepsilon$$

where *Q*_HOMO_ is the overlap area between the HOMO and d-orbitals, and *Q*_HOMO–1_ is the overlap area between the HOMO – 1 and d-orbitals. The *n*_d,up_(ε), *n*_HOMO,up_(ε), and *n*_HOMO–1,up_(ε) are the density of states (spin up) for the d-orbital of the metal and the HOMO and HOMO – 1 of the molecules, respectively. The definitions are similar for the terms of spin down. ε is the energy level.

Up to this point, it can be concluded that the overlap between the HOMO/HOMO – 1 and metal d-orbitals as well as the d-band center of M_1_ is closely related to Δ*E*_ads_. Nevertheless, these physical quantities have to be calculated, which is not convenient for an efficient assessment of the selective adsorption of α,β-UALs. Alternatively, the design of catalysts would be greatly accelerated if the basic physical properties of the catalyst could be directly correlated with selectivity,^31,32^ but this has remained a challenge in heterogeneous catalysis. Previous studies have described the correlation between the intrinsic properties of the adsorbate and the substrates using the number of electrons in the occupied d-orbital of the surface metal atom (θ_d_), the Pauling electronegativity (*E*_M_),^33,34^ and the first ionization energy (IE),^35^ thus enabling the quantitative determination of the adsorption strength of adsorbates on metals and metal oxides. As a test, we plotted the Δ*E*_ads_ of α,β-UALs adsorbed on SACs versus θ_d_ (Figures S24–S25) and *E*_M_ (Figures S26–S27), but the results clearly showed that θ_d_ or *E*_M_ did not correlate well with Δ*E*_ads_.

As mentioned earlier, CE is crucial in regulating the catalytic properties of M_1_. Indeed, we previously proposed a descriptor based on inter CE to predict the selective adsorption of acrylonitrile on M_1_@C_2_N.^36^ The effect of outer CE was not considered, however, and recently has proven to be important.^37^ To comprehensively describe both the inner and outer CE of M_1_, we introduced the cutoff function of $\left(f_{c}\left(r_{ij}\right)=\frac{1}{2}\cdot \left(\cos\left(\frac{\pi \cdot r_{ij}}{r_{c}}\right)+1\right)\right)$ and the radial symmetry function of (*G*_*i*_ = ∑_*j* = 1_^*N*_atom_^ *f*_c_(*r*_*ij*_)) using interatomic distance coordinates, which are widely used in ANN.^26,38,39^ The descriptor (λ) fully considered the physicochemical properties of all atoms involved in the inner/outer CE and M_1_, along with the MOs information on α,β-UALs, which yielded the final formulation shown in eq 6.

6 $$\lambda =\left(E_{\mathrm{HOMO}}-E_{\left(\mathrm{HOMO}-1\right)}\right)\cdot \theta_{d}\cdot \mathrm{IE}\cdot \left[E_{M}+\overset{n}{\underset{i=1}{\sum }}\overset{m+1}{\underset{j=1}{\sum }}\frac{E_{j}}{2}\cdot \left(\cos\left(\frac{\pi \cdot r_{ij}}{r_{c}}\right)+1\right)\right]\cdot 10^{-6}$$

where *E*_HOMO_ and *E*_HOMO–1_ are the DFT-calculated HOMO and HOMO – 1 energies of α,β-UALs, *n* is the number of adjacent atoms of the metal atom, *m* +1 is the number of adjacent atoms of atom *i* including itself, *E*_M_ is the electronegativity of the center atom, *E*_*j*_ is the electronegativity of the adjacent atom *j* of atom *i*, *r*_*ij*_ is the distance between atom *i* and the adjacent atom *j*, and *r*_c_ is the cutoff radius with a value of 6.0 Å used for the optimized M_1_@C_2_N and M_1_@*g*-C_3_N_4_ and 5.5 Å used for M_1_@DG and M_1_@*h*-BN, respectively. The detailed θ_d_, *E*_M_, and *E*_*j*_ with the corresponding λ are listed in Tables S6–S7.

We next attempted to disclose the relationship between λ and Δ*E*_ads_ by investigating the adsorption of C=O/C=C bonds of C_3_H_4_O (Figure 3a), C_4_H_6_O (Figure 3b), C_5_H_8_O (Figure 3c), and 2-pentenal (Figure 3d) on the four SACs. As a result, Δ*E*_ads_ and λ are found to correlate well and can be expressed by the following equation:

7 $$\Delta E_{\mathrm{ads}}=k\lambda +b$$

where *k* is the slope and *b* is the intercept. We found that the *k* value depends on the molecular structure of α,β-UAL; that is, different α,β-UALs have different sensitivities to the CE. C_3_H_4_O, C_4_H_6_O, C_5_H_8_O, and 2-pentenal have *k* values of 0.12, 0.14, 0.17, and 0.15, suggesting that the adsorption of C_5_H_8_O is most sensitive to the CE of SACs. In addition, Figure S28a shows that the value of *E*_HOMO_ – *E*_HOMO–1_ correlates well with *k* with an *R* ^2^ of 0.96. We have also calculated the HOMO and HOMO – 1 energies of these molecules at the B3LYP/6-31g level,^40^ and the corresponding energies are given in Table S8. It can be seen that the trend of the *E*_HOMO_ – *E*_HOMO–1_ values for these α,β-UAL molecules does not change. Furthermore, Figure S28b shows a good linear relationship between the *E*_HOMO_ – *E*_(HOMO–1)_ obtained by the two different methods with *R* ^2^ = 0.98. These test results clearly show that the use of other calculation methods does not change the order of *k*, i.e., does not change our conclusions in this work. For the *ald*/*ene*-mode on each SAC (M_1_@C_2_N (Figure 3e), M_1_@*g*-C_3_N_4_ (Figure 3f), M_1_@DG (Figure 3g), and M_1_@*h*-BN (Figure 3h)), the plot of Δ*E*_ads_ versus λ still exhibits excellent linearity.

**Figure 3:**
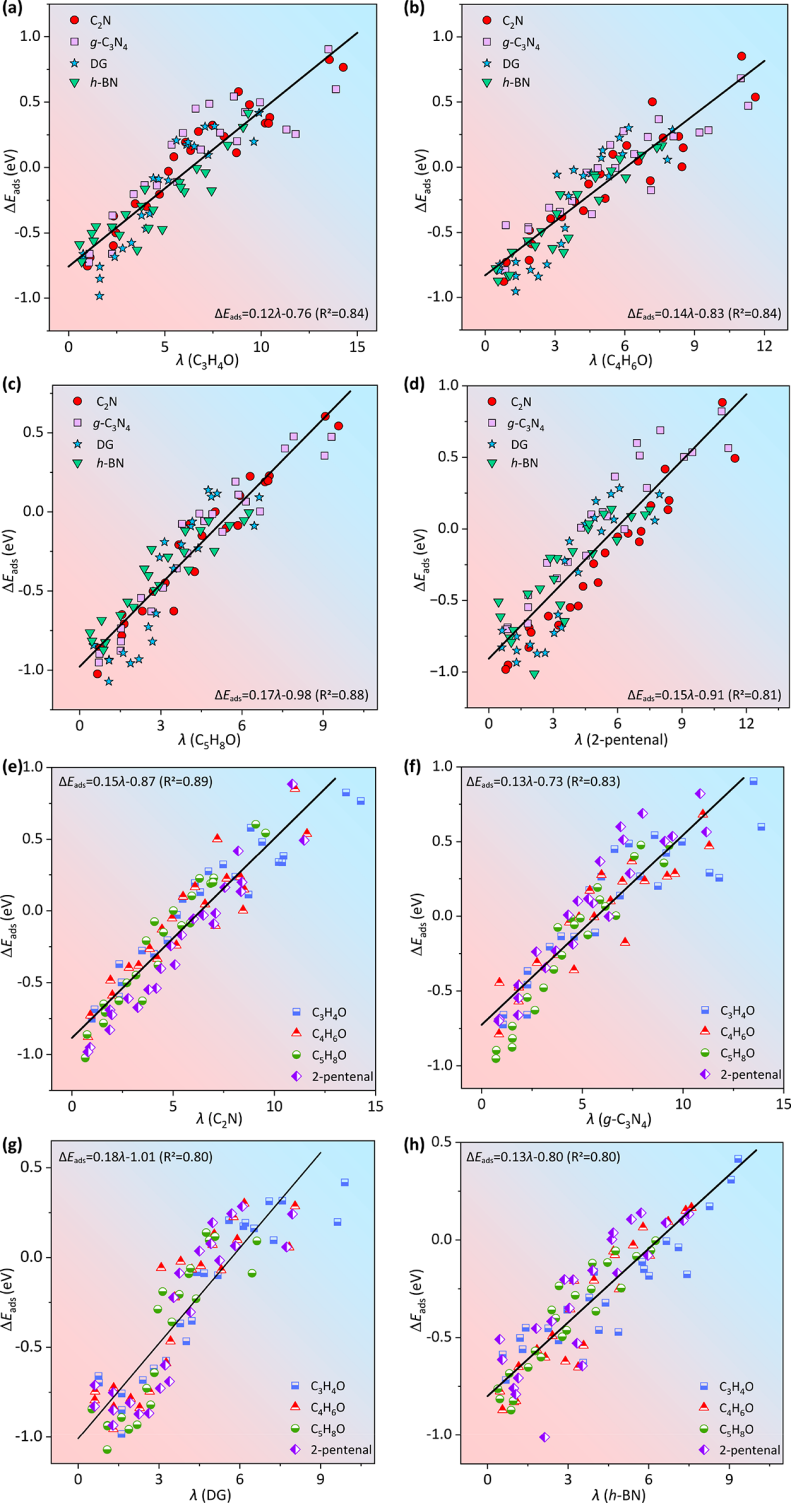
Δ*E*_ads_ of four α,β-UALs as a function of descriptor (λ) on M_1_@C_2_N, M_1_@*g*-C_3_N_4_, M_1_@DG, and M_1_@*h*-BN: (a) C_3_H_4_O, (b) C_4_H_6_O, (c) C_5_H_8_O, and (d) 2-pentenal. Δ*E*_ads_ of C_3_H_4_O, C_4_H_6_O, C_5_H_8_O, and 2-pentenal as a function of λ on (e) M_1_@C_2_N, (f) M_1_@*g*-C_3_N_4_, (g) M_1_@DG, and (h) M_1_@*h*-BN.

The results show that λ always works well, regardless of the same α,β-UAL adsorbed on different SACs or different α,β-UALs adsorbed on the same SAC. Larger λ indicates that the *ene*-mode dominates when the C=C bond has a greater adsorption strength on the SAC, whereas a smaller λ favors the *ald*-mode. Note that for the IB subgroup (Cu, Ag, and Au), the selective adsorption of α,β-UALs is poorly described (Figures S29–S30), which may be attributed to their weak adsorption abilities to molecules caused by the complete filling of the d-orbitals and the low position of the d-band center relative to the Fermi energy level.^41,42^ Furthermore, the selective adsorption of α,β-UALs cannot be well described if the outer CE was not considered (Figures S31–S32), suggesting the importance of the peripheral CE in quantitatively understanding the selective interactions of α,β-UALs on 2D SACs.

A schematic workflow of the descriptor is depicted in Figure 4a. The cutoff and symmetry functions were adopted to construct λ, fully considering the peripheral CE effect. The inputs to the descriptor consist of interpretable physical parameters (e.g., θ_d_, IE, *E*_M_, and *r*_*ij*_), while the output contains the unequivocal relationship between the electronic d-band center or Bader charge^41,43^ and the value of $\left(\theta_{d}\cdot \mathrm{IE}\cdot \left[E_{M}+\overset{n}{\underset{i=1}{\sum }}\overset{m+1}{\underset{j=1}{\sum }}\frac{E_{j}}{2}\cdot \left(\cos\left(\frac{\pi \cdot r_{\mathrm{ij}}}{r_{c}}\right)+1\right)\right]\cdot 10^{-6}\right)$ in λ (Figures S33–S34). Finally, to demonstrate the universality of λ, the adsorption of C_3_H_4_O on M_1_-doped C_2_N (Figure 4b), the M_1_@*h*-BN with N vacancies (M_1_@*h*-BN (B_v_)) (Figure 4c), and the adsorption of isophorone on M_1_@C_2_N (Figure 4d with the HOMO and HOMO – 1 of isophorone) were investigated. Figures 4e and 4f show that Δ*E*_ads_ for the C_3_H_4_O adsorbed on M_1_-doped C_2_N and isophorone adsorbed on M_1_@C_2_N are well predicted by the equations in Figures 3a and 3e, respectively. Encouragingly, even for a new system of the isophorone adsorbed on M_1_@*h*-BN (B_v_), λ still shows good correlation with the DFT-calculated Δ*E*_ads_ (Figure 4g). Detailed data are presented in Table S9.

**Figure 4:**
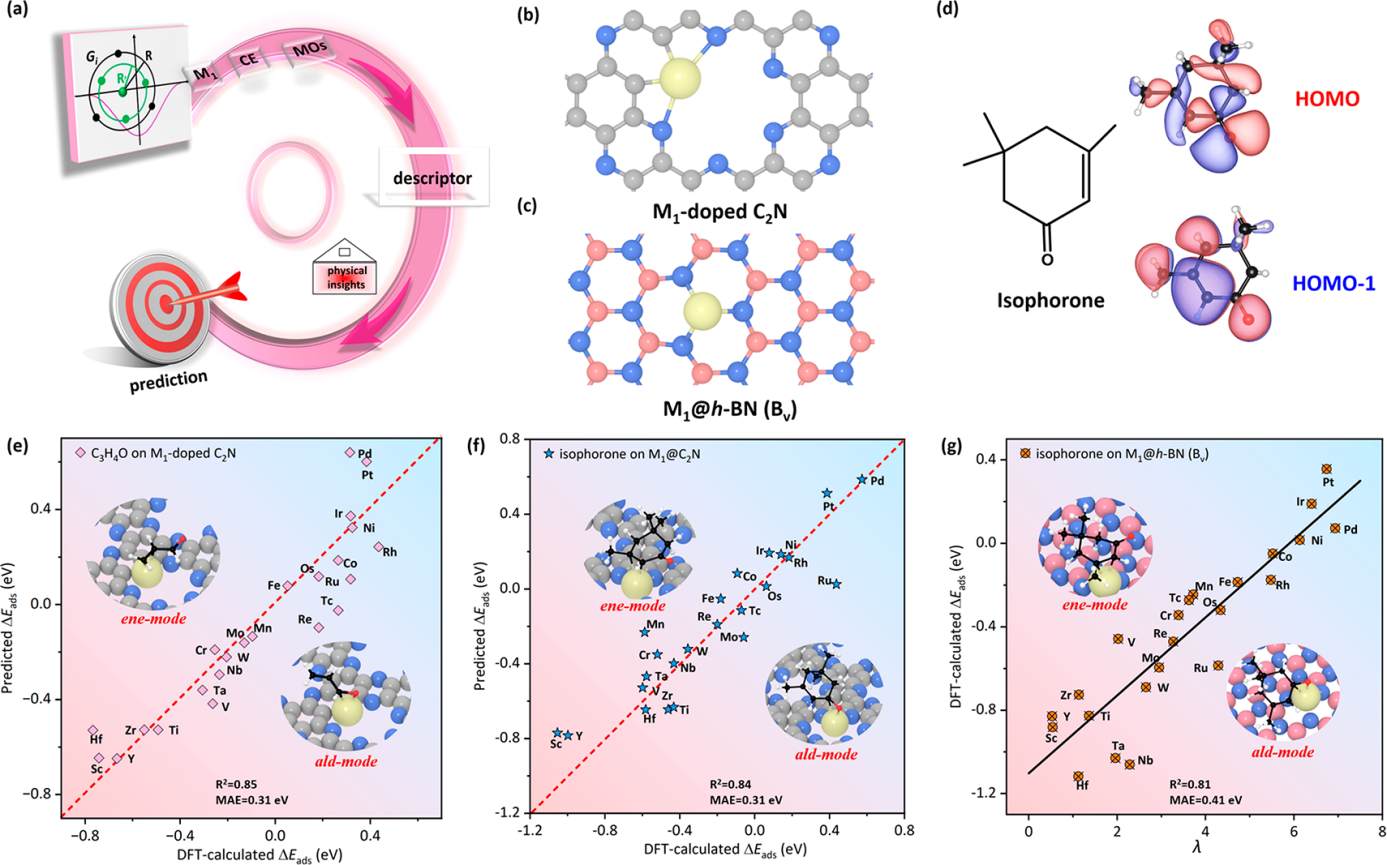
(a) Workflow of the descriptor, (b) M_1_-doped C_2_N, (c) M_1_@*h*-BN (B_v_), and (d) isophorone with HOMO and HOMO – 1. (e) Comparison of the predicted and DFT-calculated Δ*E*_ads_ for C_3_H_4_O adsorbed on M_1_-doped C_2_N and (f) for isophorone adsorbed on M_1_@C_2_N. (g) DFT-calculated Δ*E*_ads_ of isophorone as a function of λ on M_1_@*h*-BN (B_v_). Yellow: metal; blue: N; gray: C in support; pink: B; white: H; black: C in isophorone; red: O.

## 4 Conclusions

In summary, we extended the application of frontier MO theory to understand the selective interactions over 2D SACs. A descriptor was developed via combining the HOMO/HOMO – 1 of α,β-UALs with the fundamental physical properties of SACs that consider both the inner and outer CEs, which are precisely described by the radial symmetry functions. This descriptor allows a fast and quantitative description of the selective adsorption of the C=O/C=C bonds of α,β-UALs on 2D SACs. Moreover, all the parameters in this descriptor are readily accessible and interpretable. This work provides not only guidance for the design of 2D catalysts for the selective hydrogenation of α,β-UALs to α,β-UOLs but also definite physical quantities for constructing more comprehensive machine learning models in the future.

## Supplementary Material



## Notes

Structures of 2D materials supported SACs and adsorption of C_3_H_4_O in this work; PDOS of catalysts and MODOS of molecules; 2D contour diagrams of Δ*E*_ads_ of molecules as a functional of the d-band center of M_1_; relationships between θ_d_ (*E*_M_) and Δ*E*_ads_ and between *E*_HOMO_ – *E*_HOMO–1_ and slope *k*; calculated *E*_HOMO_ – *E*_(HOMO–1)_; relationship between descriptor λ and Δ*E*_ads_; relationship between Bader charge/d-band center of M_1_ and λ′; adsorption energies and MOs energies; detailed physical parameters; λ values for different models; adsorption energies and λ values corresponding to predicted structures (PDF)
