# Supplementary material for: Peripheral Coordination-Dependent Descriptor for Selective Interactions between Near-Frontier Molecular Orbitals and Single-Atom Catalysts
Source: Precis Chem. 2023 Apr 13;1(7):429–36. doi: 10.1021/prechem.3c00015 (PMC12382409; doi:10.1021/prechem.3c00015)
Supplement: Supplementary file 1 [file pc3c00015_si_001.pdf]

Supporting Information

Peripheral Coordination-Dependent Descriptor for Selective Interactions between Near-Frontier Molecular Orbitals and Single-Atom Catalysts

Bingqing Ge [a,b,+], *Fenfei Wei* [a,+], *Qiang Wan* [a], *Hongwei Zhang* [b], *Pei Yuan* *[,b], *Sen Lin* *[, a]

[a]State Key Laboratory of Photocatalysis on Energy and Environment, College of Chemistry, Fuzhou University, Fuzhou 350002, China

[b]National Engineering Research Center of Chemical Fertilizer Catalyst, College of Chemical Engineering, Fuzhou University, Fuzhou 350002, China

[+]These authors contributed equally to this work.

*Corresponding author. Email: slin@fzu.edu.cn and yuanpei@fzu.edu.cn

Contents

1. Supporting figures…………………………………………………………....3

2. Supporting tables……………………………………………………………37

1. Supporting figures

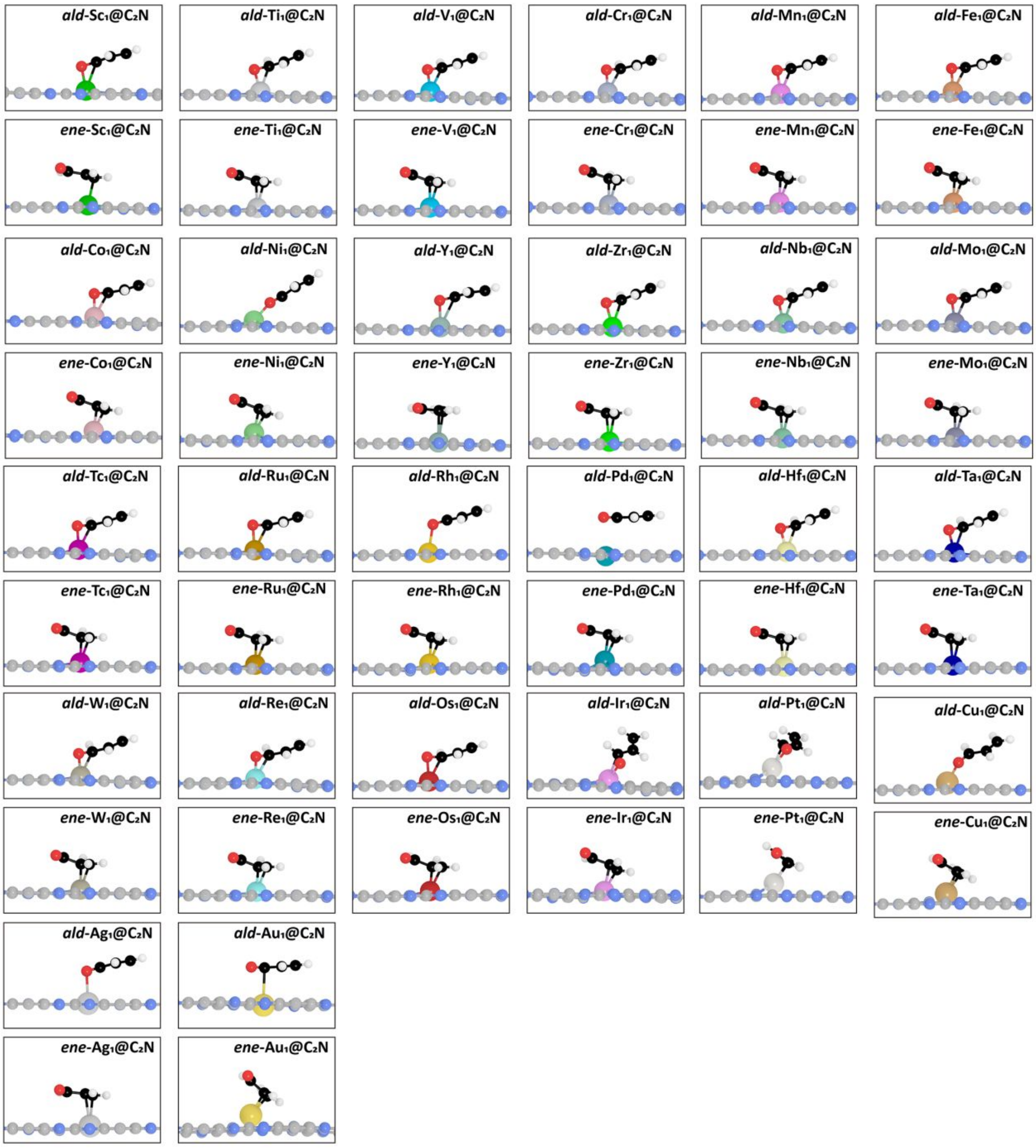

Figure S1. The configurations for the *ald*-mode and *ene*-mode of acrolein (C_3H_4O) adsorbed on M_1@C_2N. Color scheme: Gray: C atom of C_2N; Blue: N atom of C_2N; White: H atom of C_3H_4O; Black of C_3H_4O: C atom of C_3H_4O; Red: O atom of C_3H_4O.

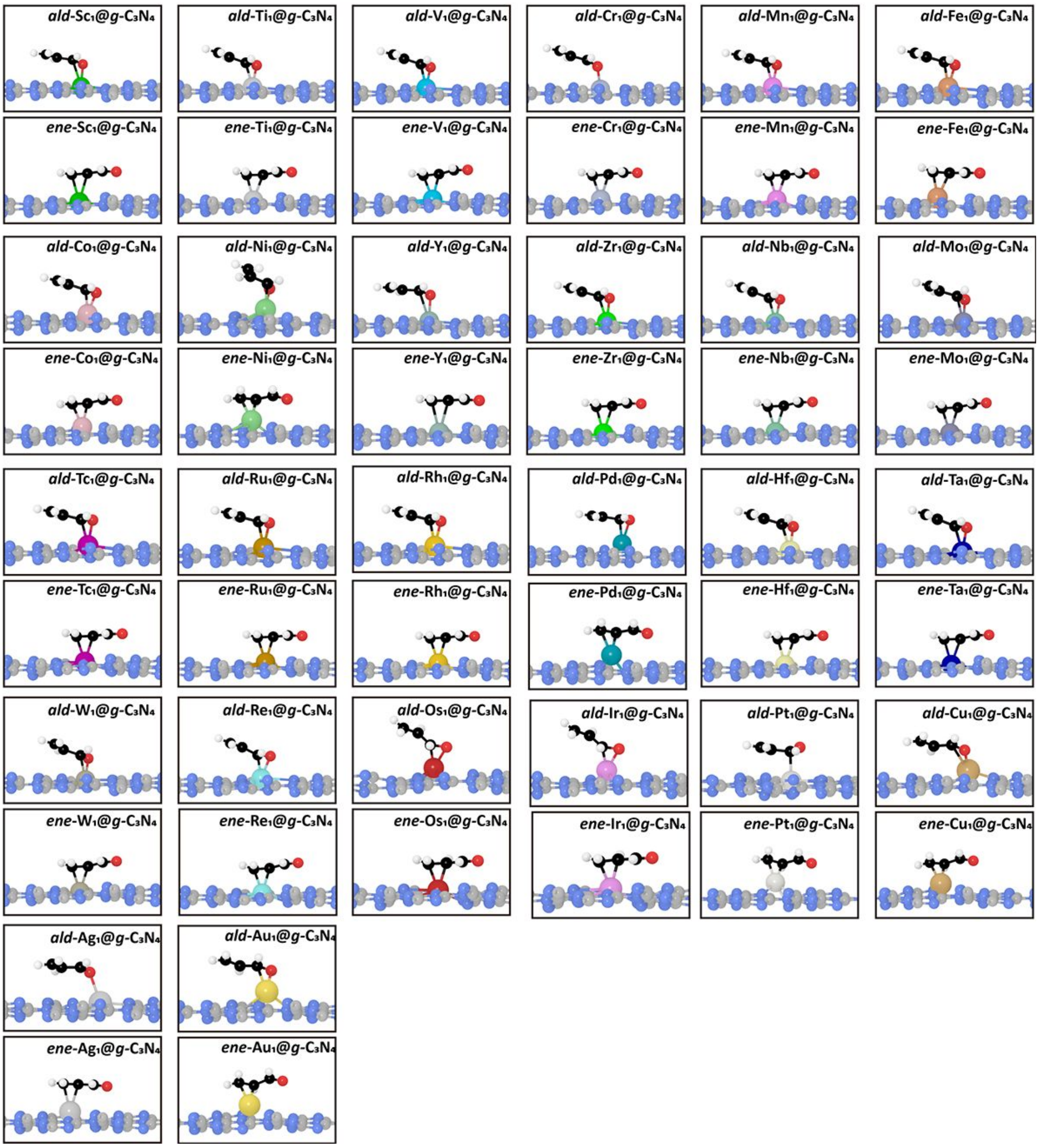

Figure S2. The configurations for the *ald*-mode and *ene*-mode of C_3H_4O adsorbed on M_1@*g*-C_3N_4. Color scheme: Gray: C atom of C_2N; Blue: N atom of *g*-C_3N_4; White: H atom of C_3H_4O; Black of C_3H_4O: C atom of C_3H_4O; Red: O atom of C_3H_4O.

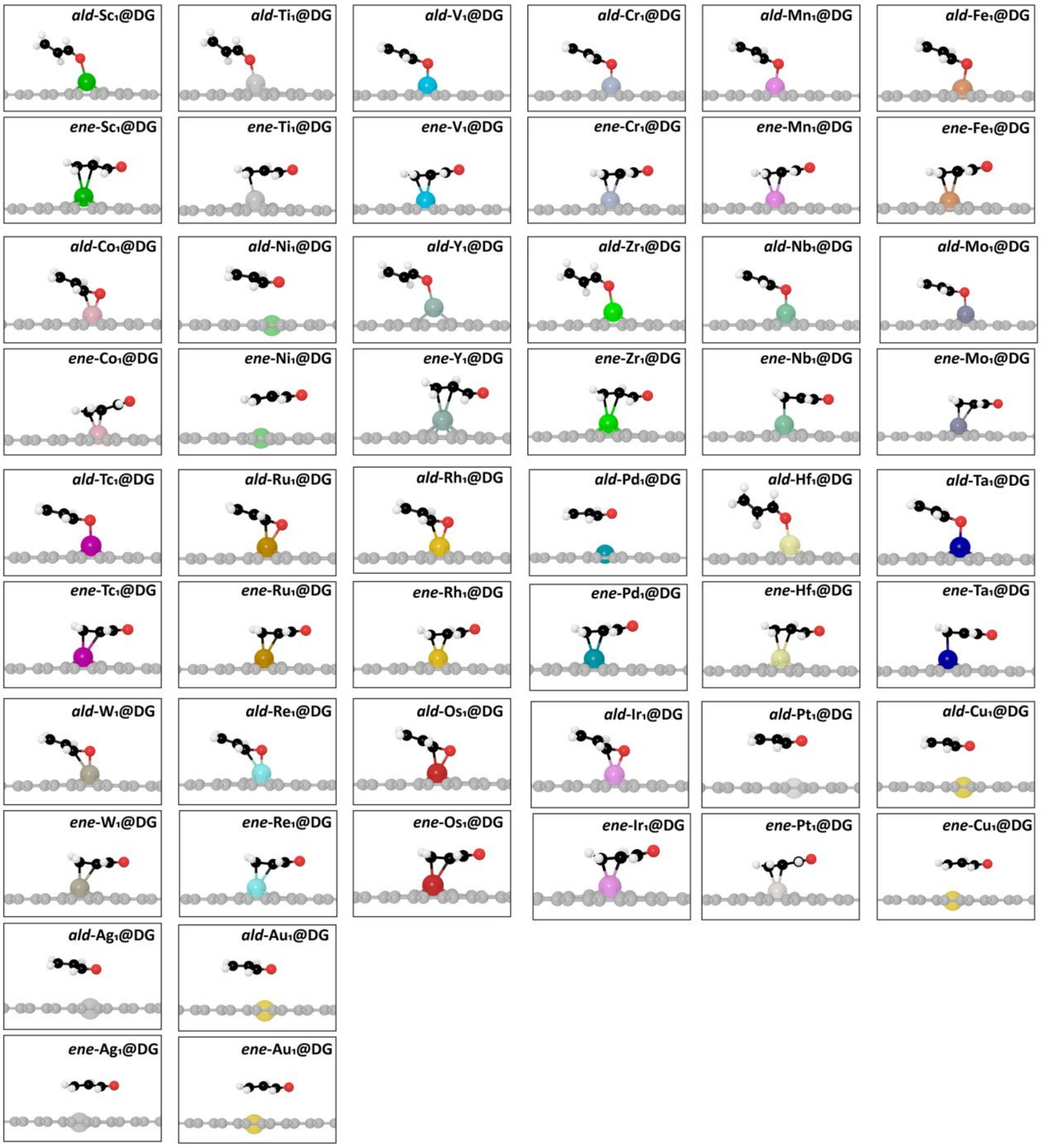

Figure S3. The configurations for the *ald*-mode and *ene*-mode of C_3H_4O adsorbed on M_1@DG. Color scheme: Gray: C atom of DG; White: H atom of C_3H_4O; Black of C_3H_4O: C atom of C_3H_4O; Red: O atom of C_3H_4O.

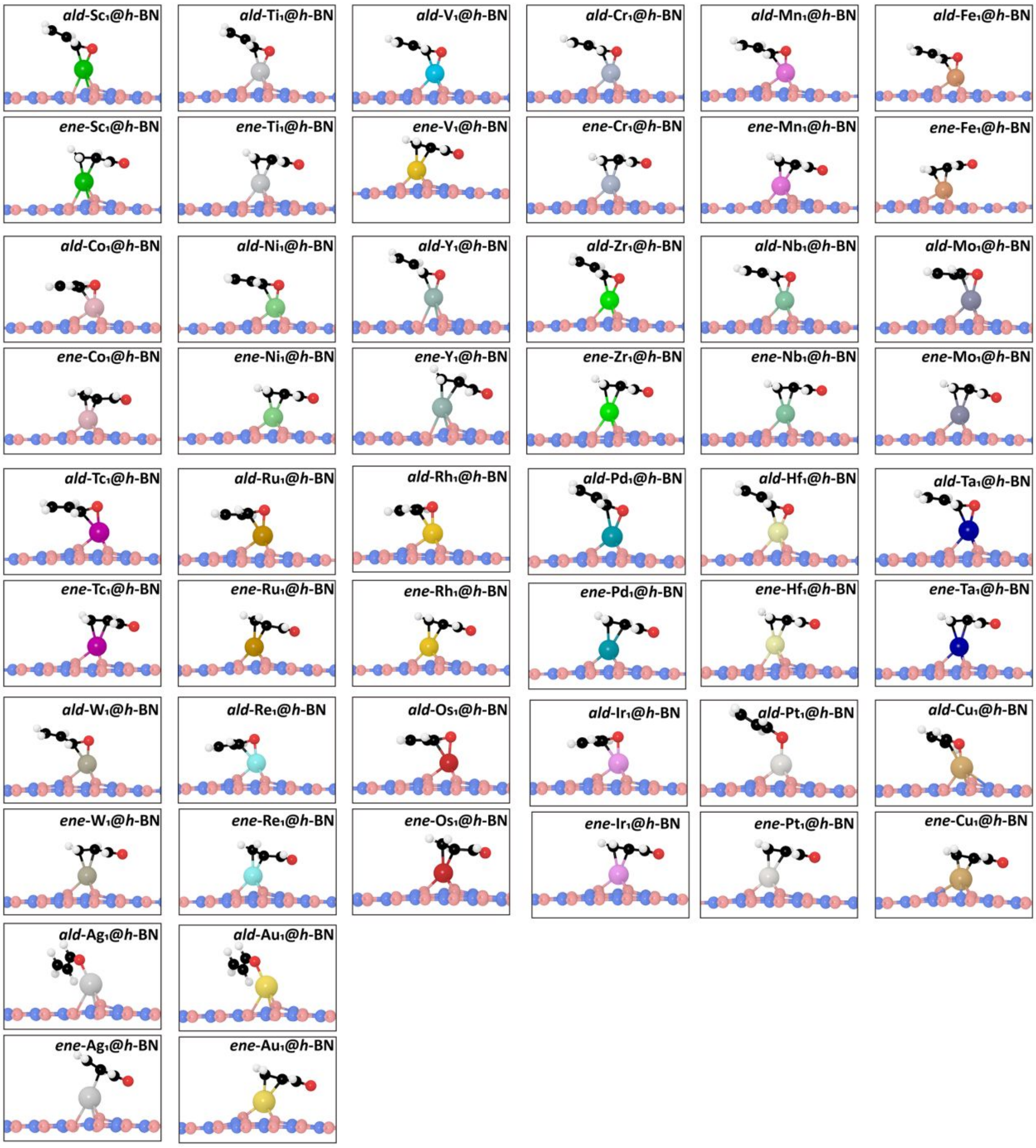

Figure S4. The configurations for the *ald*-mode and *ene*-mode of C_3H_4O adsorbed on M_1@*h*-BN. Color scheme: Pink: B atom; Blue: N atom of *h*-BN; White: H atom of C_3H_4O; Black of C_3H_4O: C atom of C_3H_4O; Red: O atom of C_3H_4O.

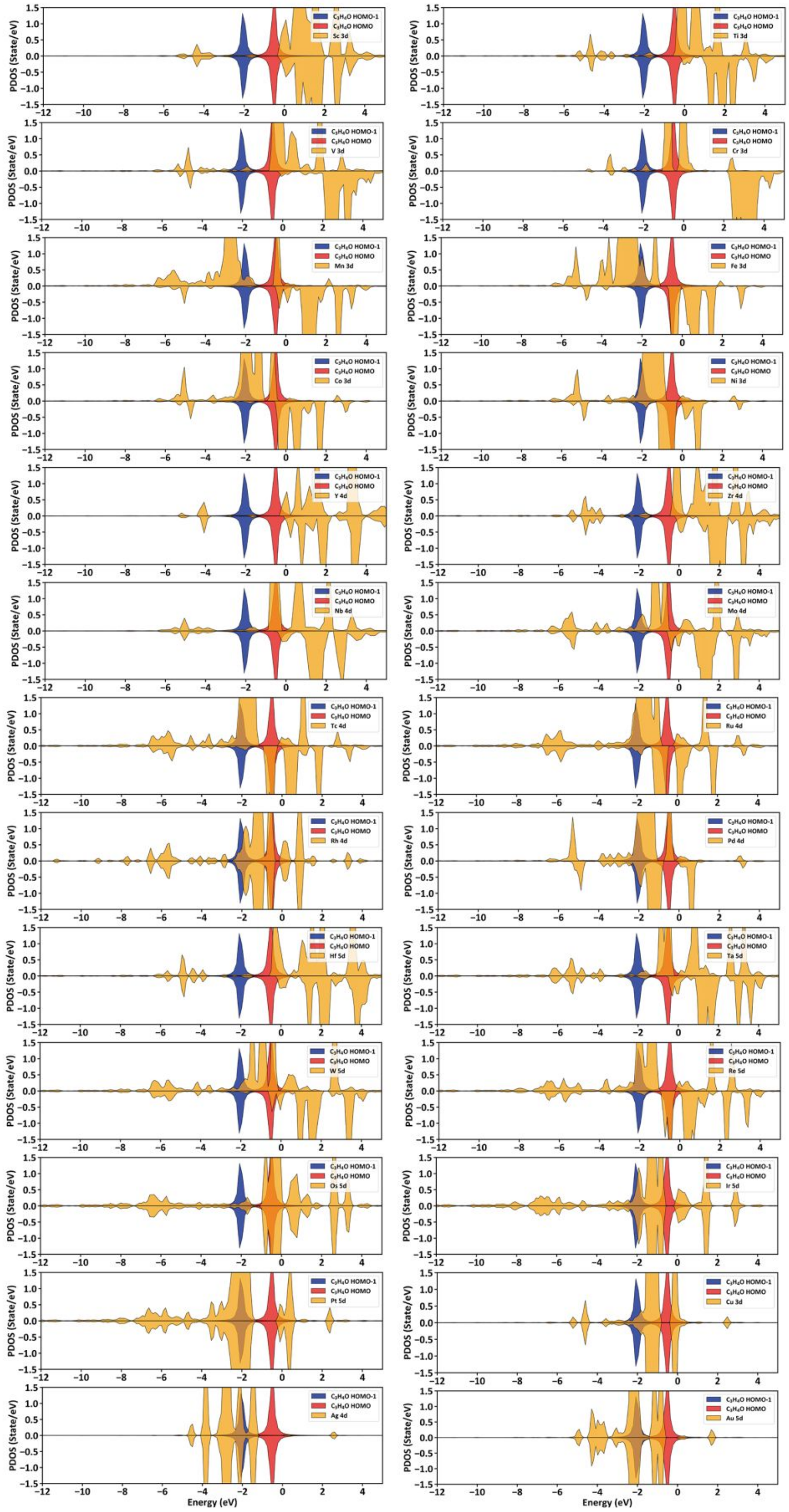

Figure S5. The overlap between the MOs (HOMO, red area, HOMO-1, blue area) of C_3H_4O and the PDOS of the *d*-states (yellow area) of M_1 on pristine clean $M_1@C_2N$.

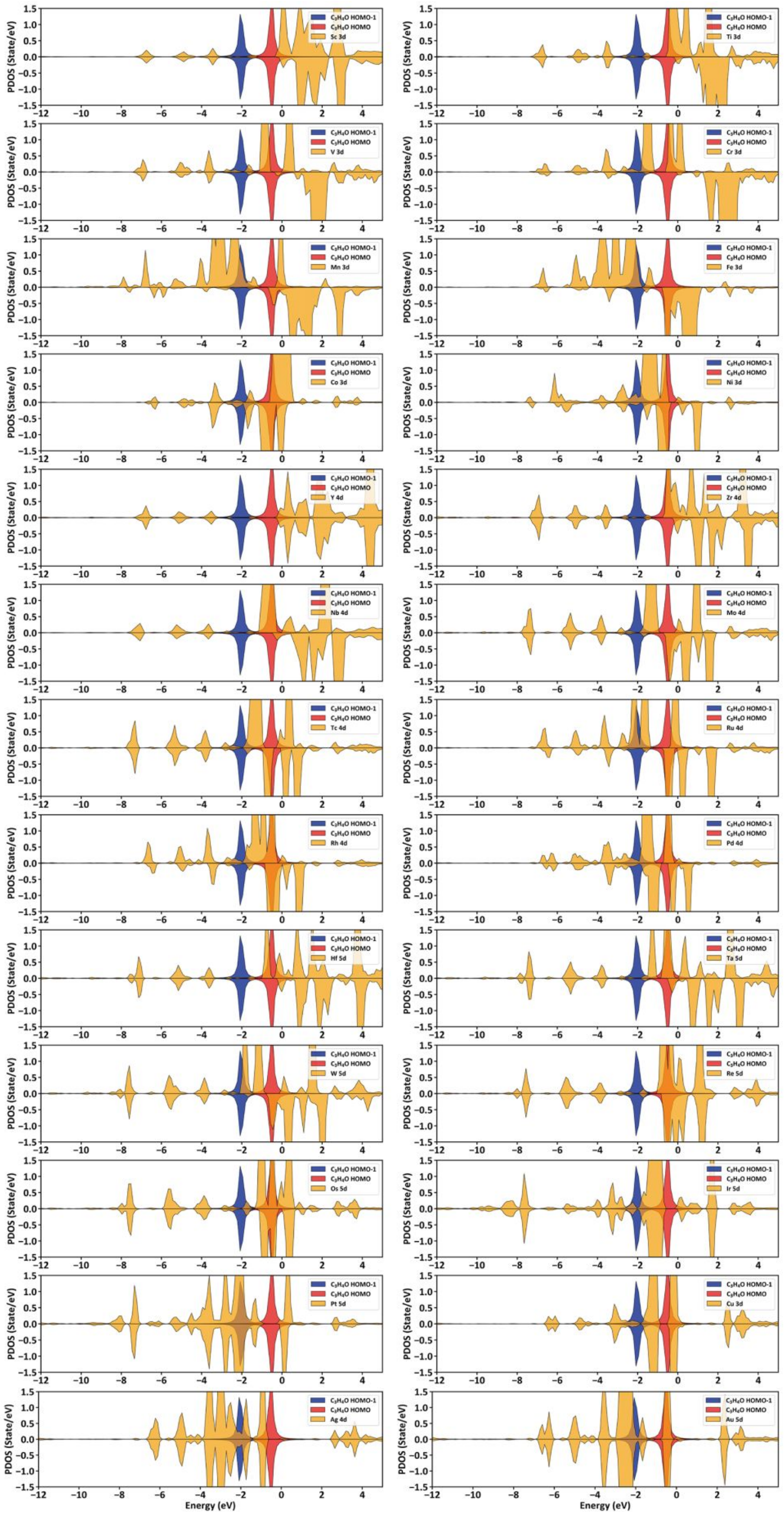

Figure S6. The overlap between the MOs (HOMO, red area, HOMO-1, blue area) of C_3H_4O and the PDOS of the *d*-states (yellow area) of M_1 on pristine M_1@*g*-C_3N_4.

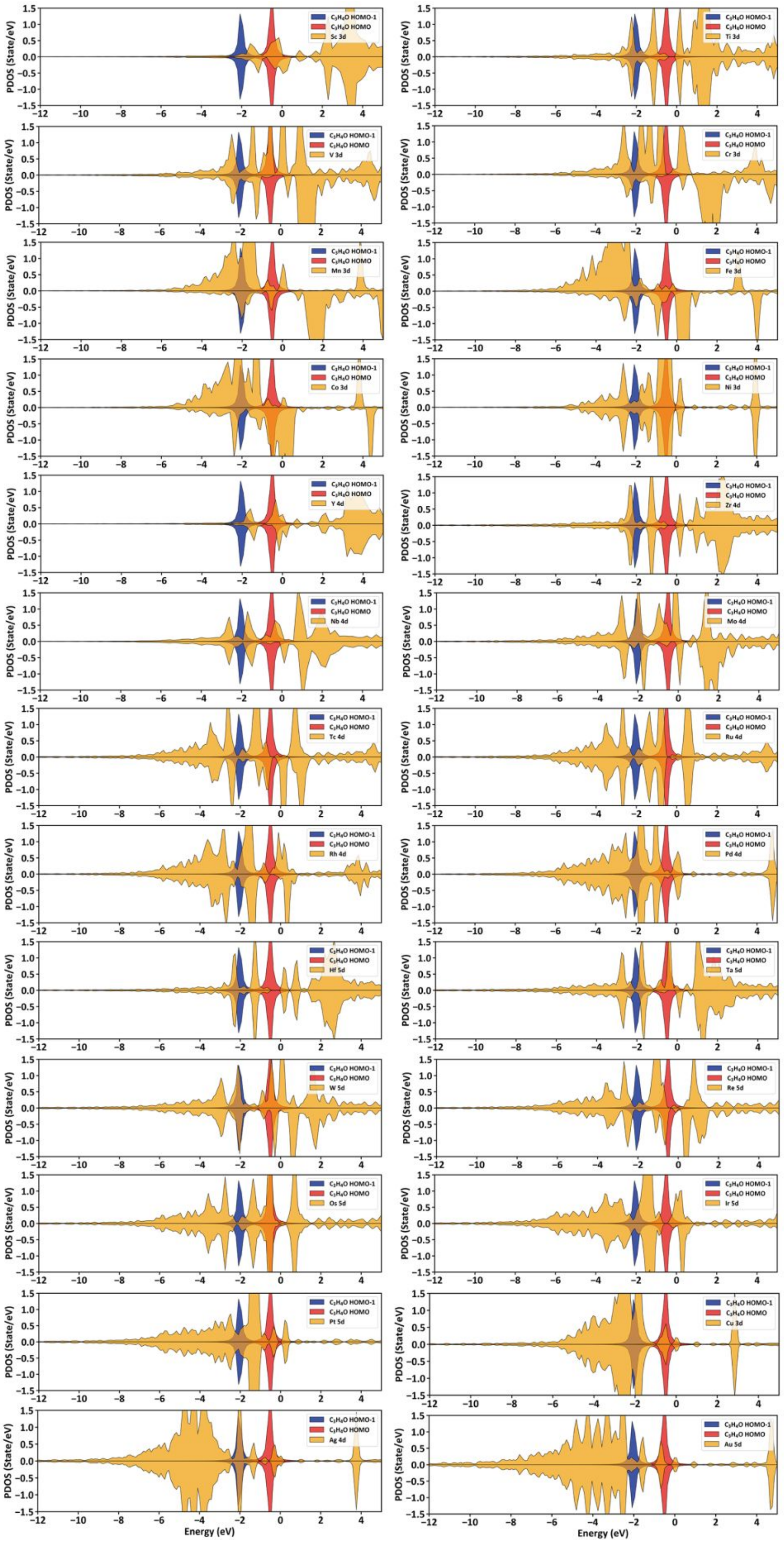

Figure S7. The overlap between the MOs (HOMO, red area, HOMO-1, blue area) of C_3H_4O and the PDOS of the *d*-states (yellow area) of M_1 on pristine M_1@DG.

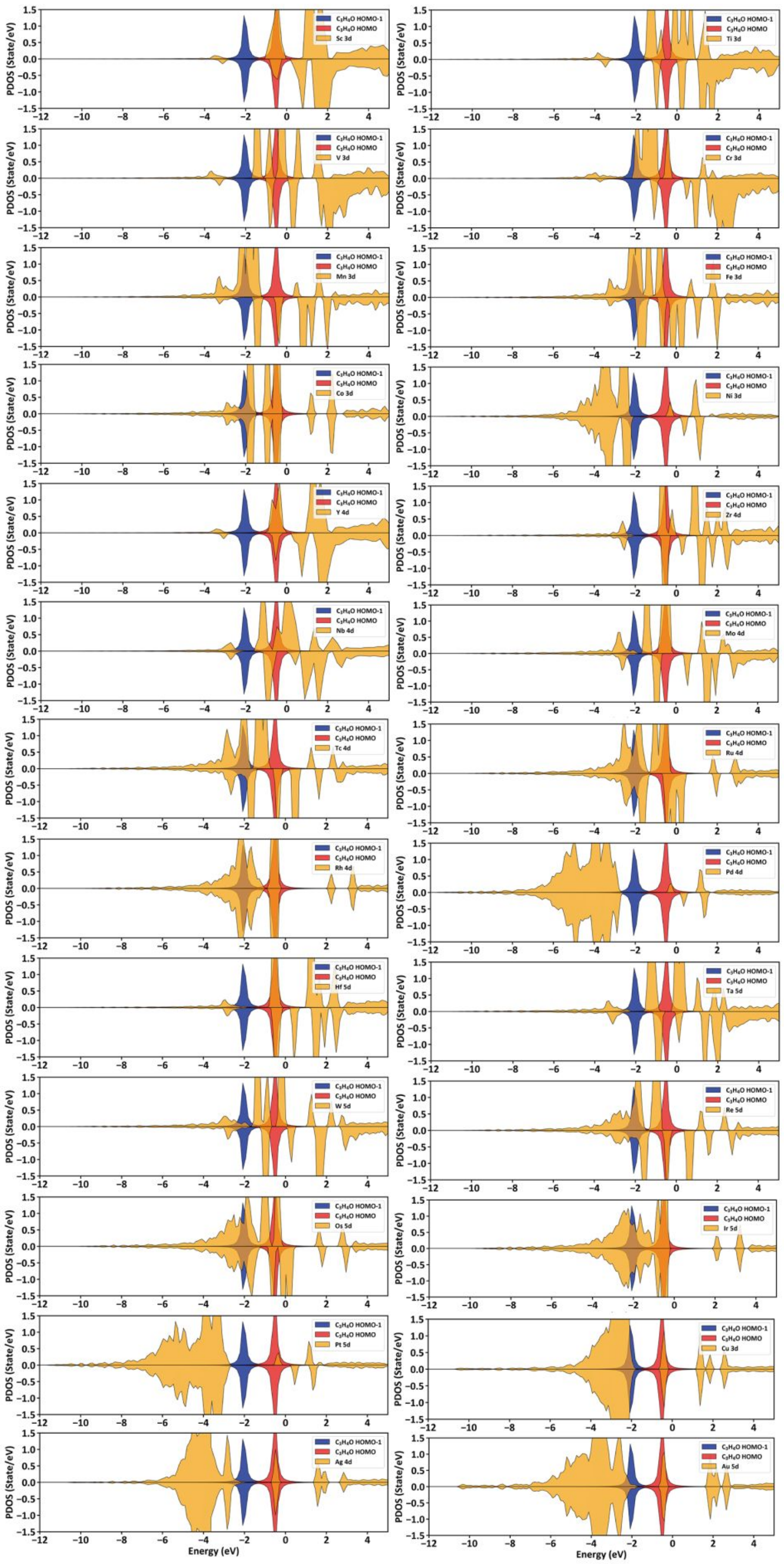

Figure S8. The overlap between the MOs (HOMO, red area, HOMO-1, blue area) of C_3H_4O and the PDOS of the *d*-states (yellow area) of M_1 on pristine M_1@*h*-BN.

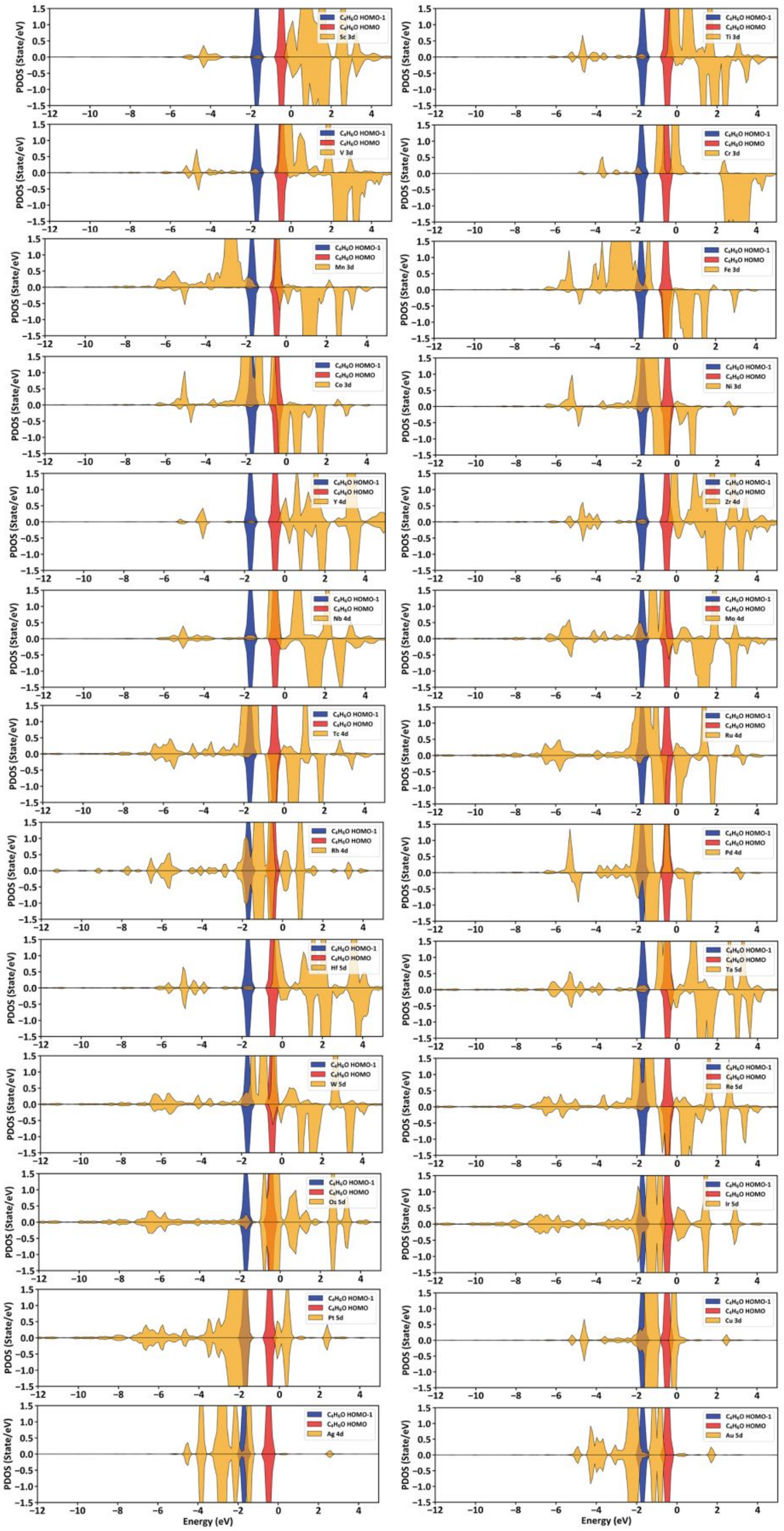

Figure S9. The overlap between the MOs (HOMO, red area, HOMO-1, blue area) of C_4H_6O and the PDOS of the *d*-states (yellow area) of M_1 on pristine $M_1@C_2N$.

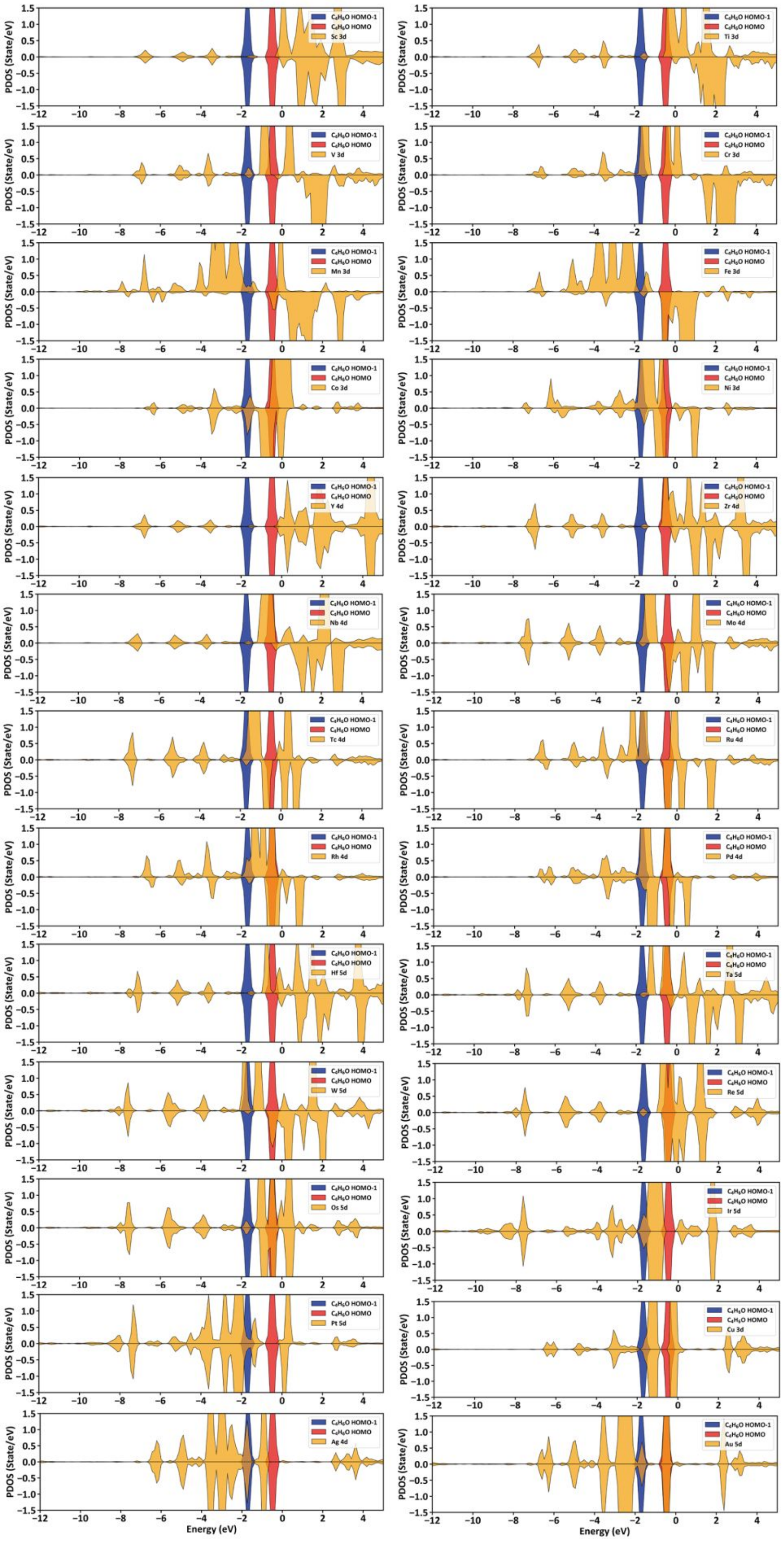

Figure S10. The overlap between the MOs (HOMO, red area, HOMO-1, blue area) of C_4H_6O and the PDOS of the *d*-states (yellow area) of M_1 on pristine M_1@*g*-C_3N_4.

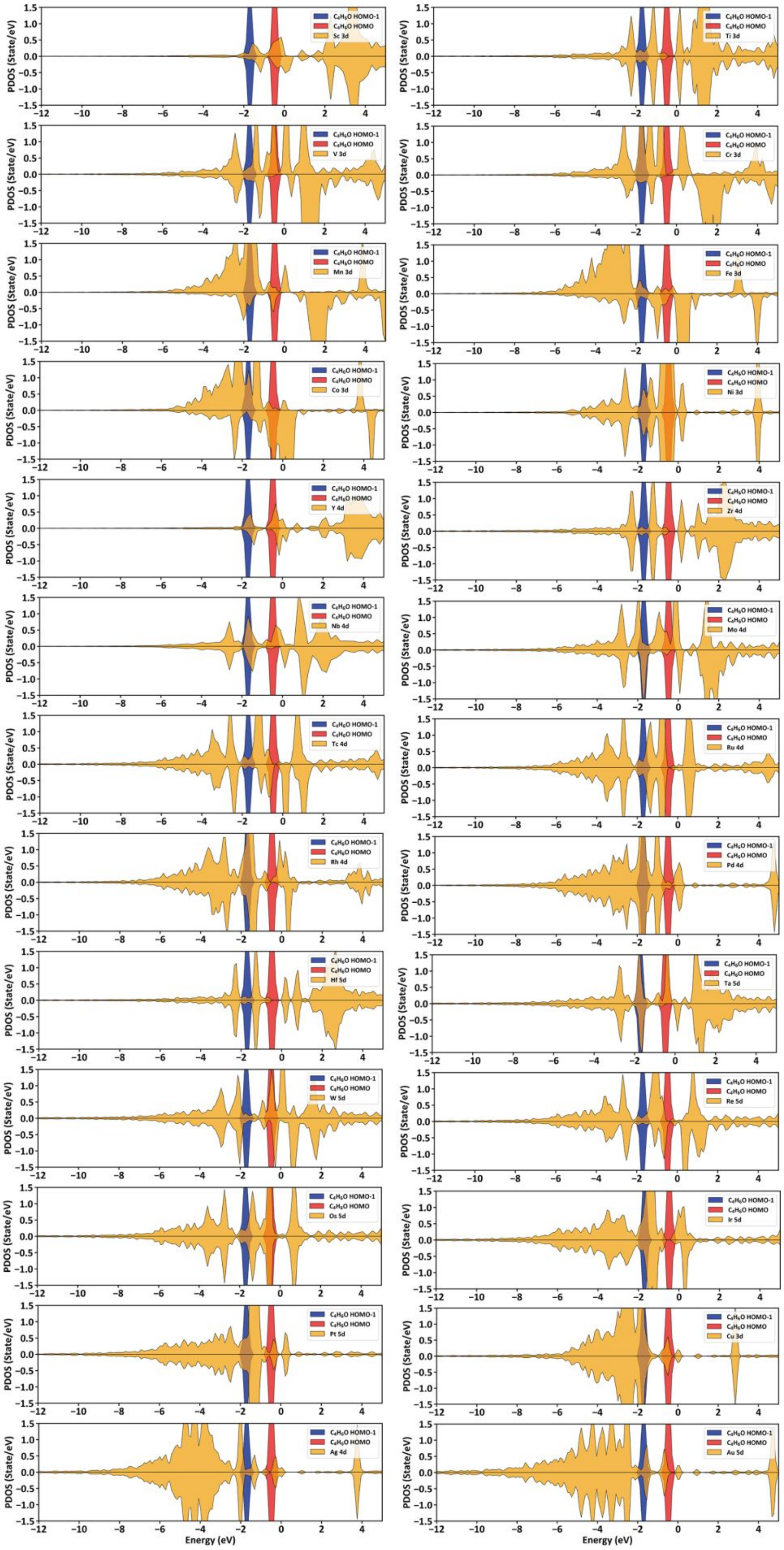

Figure S11. The overlap between the MOs (HOMO, red area, HOMO-1, blue area) of C_4H_6O and the PDOS of the *d*-states (yellow area) of M_1 on pristine M_1@DG.

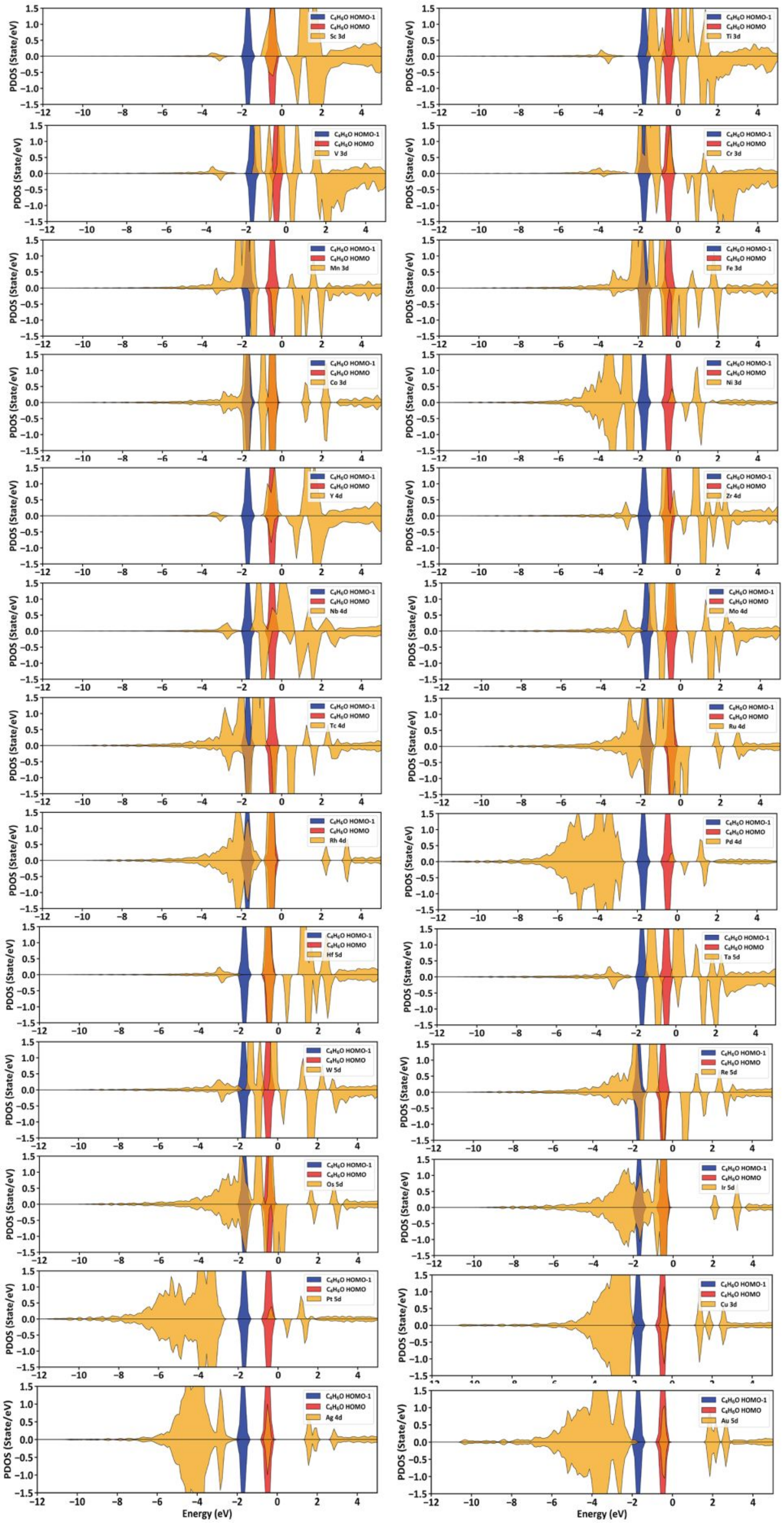

Figure S12. The overlap between the MOs (HOMO, red area, HOMO-1, blue area) of C_4H_6O and the PDOS of the *d*-states (yellow area) of M_1 on pristine M_1@*h*-BN.

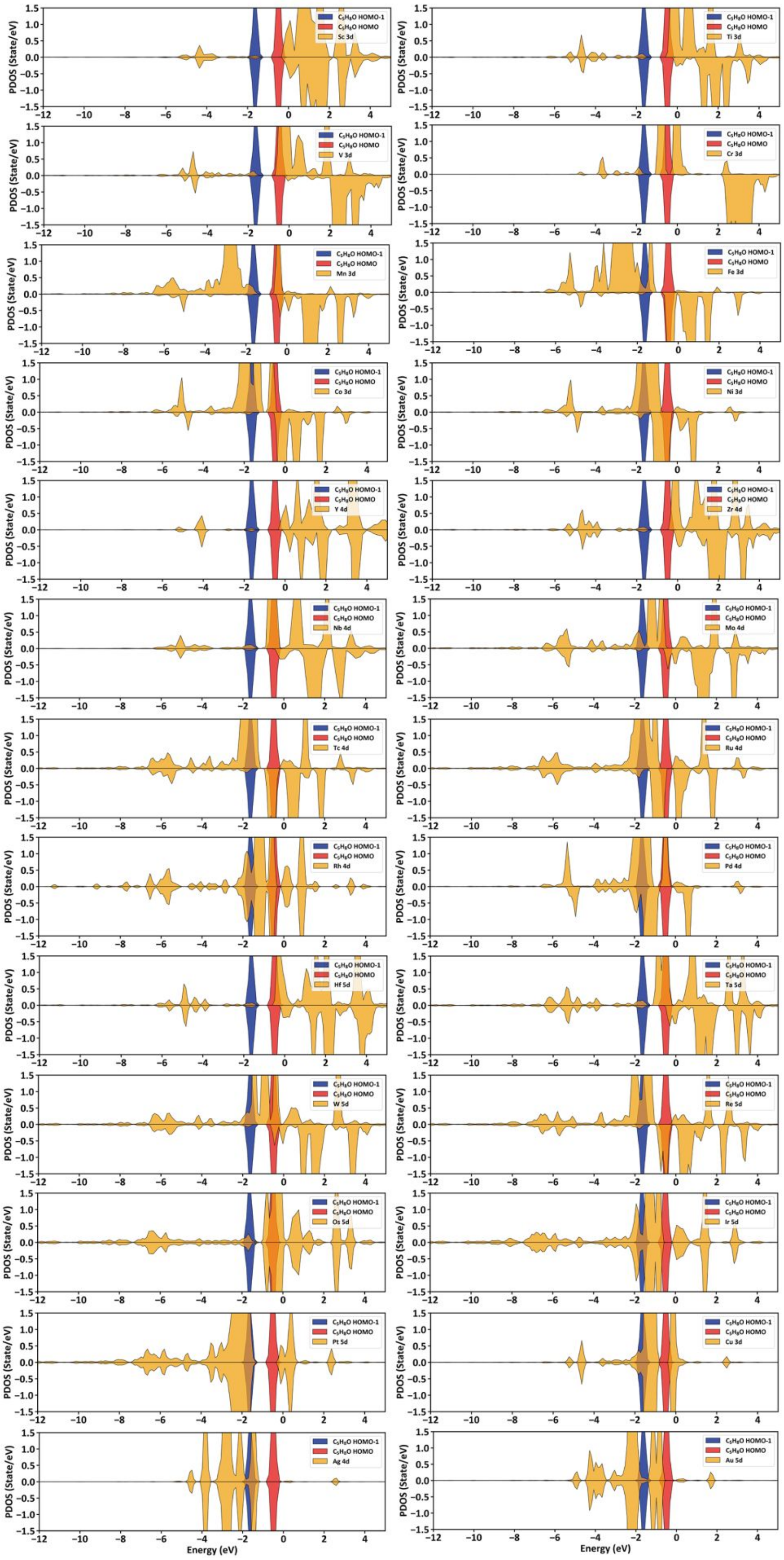

Figure S13. The overlap between the MOs (HOMO, red area, HOMO-1, blue area) of C_5H_8O and the PDOS of the *d*-states (yellow area) of M_1 on pristine $M_1@C_2N$.

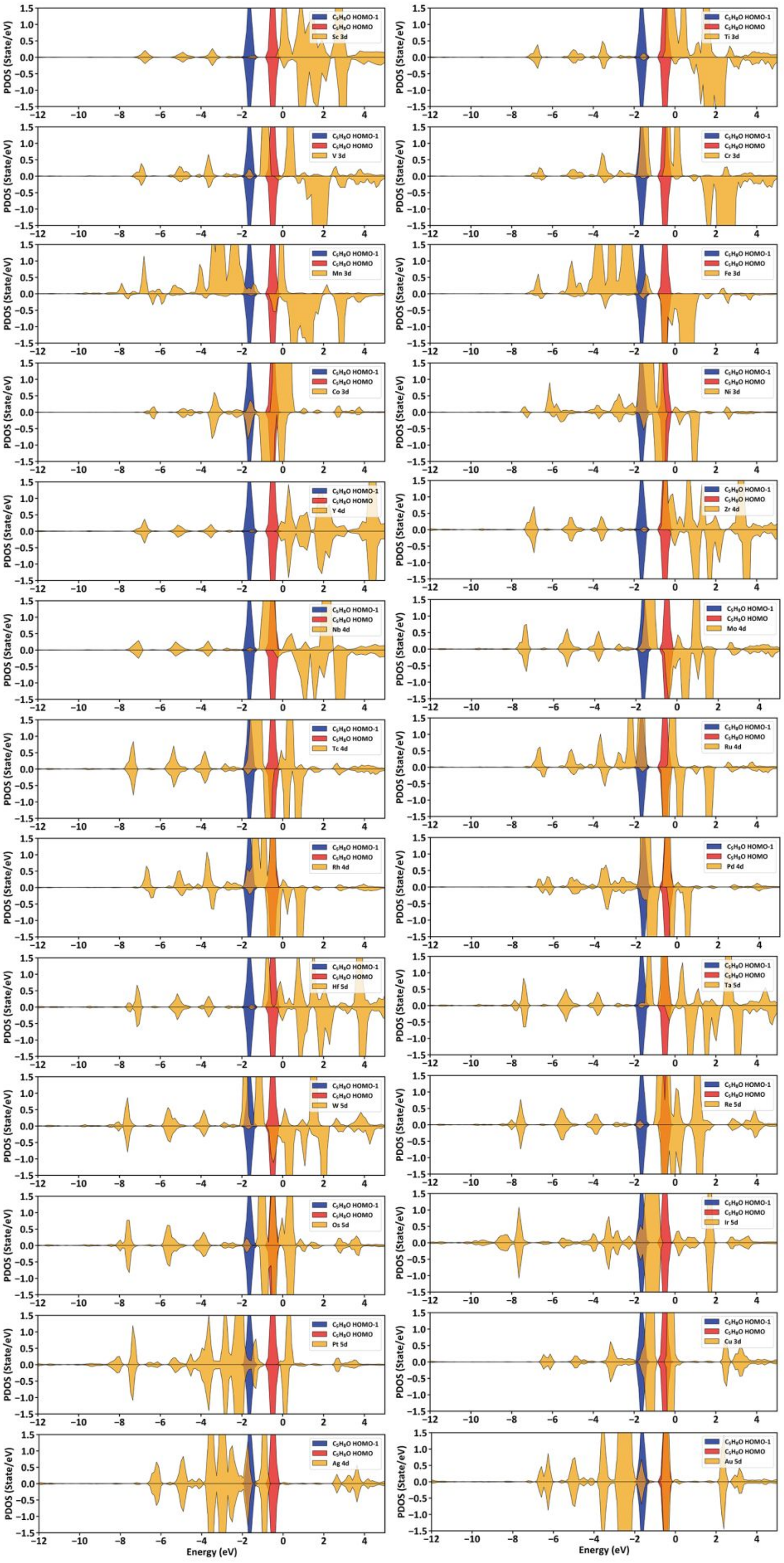

Figure S14. The overlap between the MOs (HOMO, red area, HOMO-1, blue area) of C_5H_8O and the PDOS of the *d*-states (yellow area) of M_1 on pristine M_1@*g*-C_3N_4.

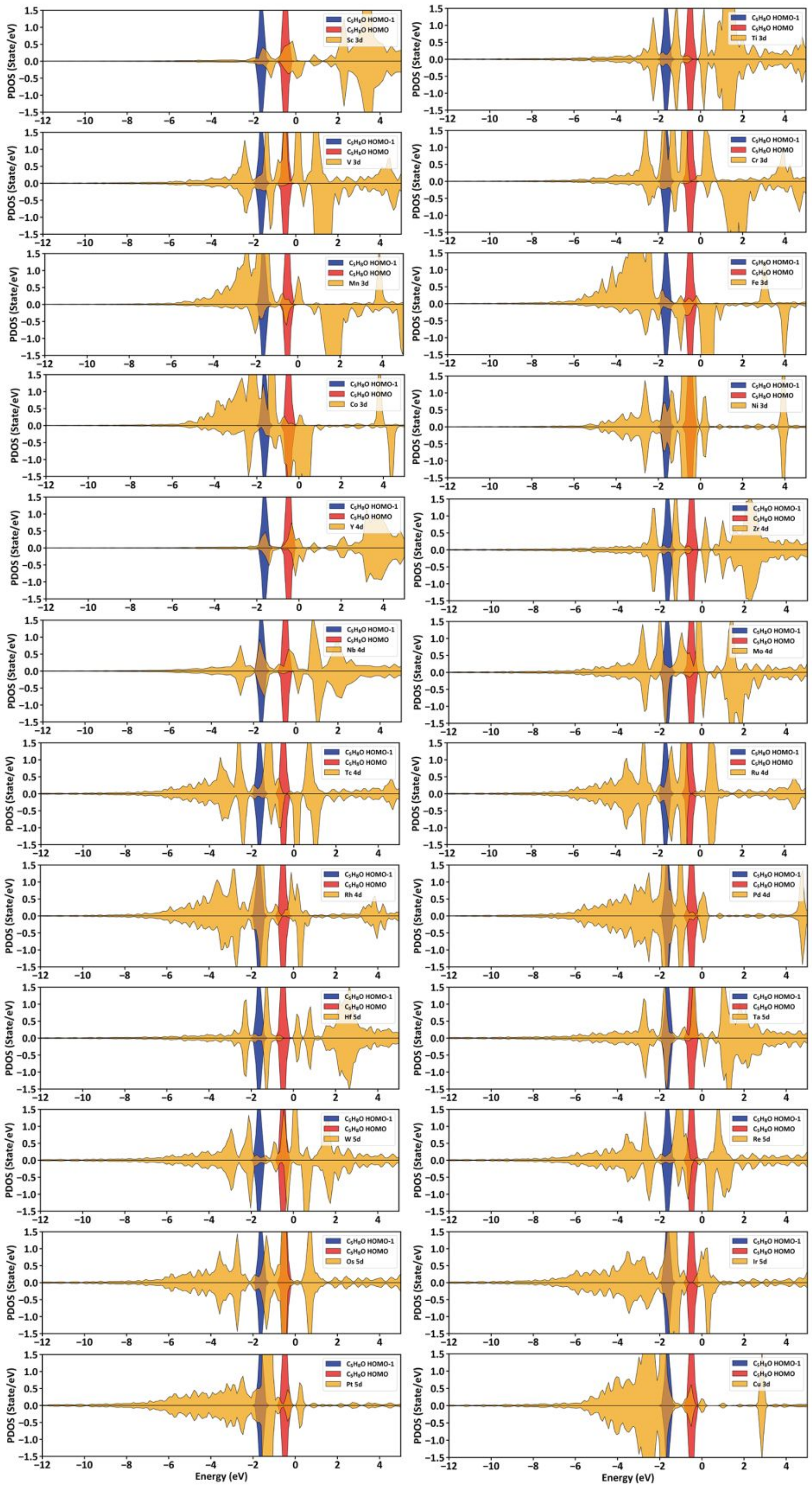

Figure S15. The overlap between the MOs (HOMO, red area, HOMO-1, blue area) of C_5H_8O and the PDOS of the *d*-states (yellow area) of M_1 on pristine M_1@DG.

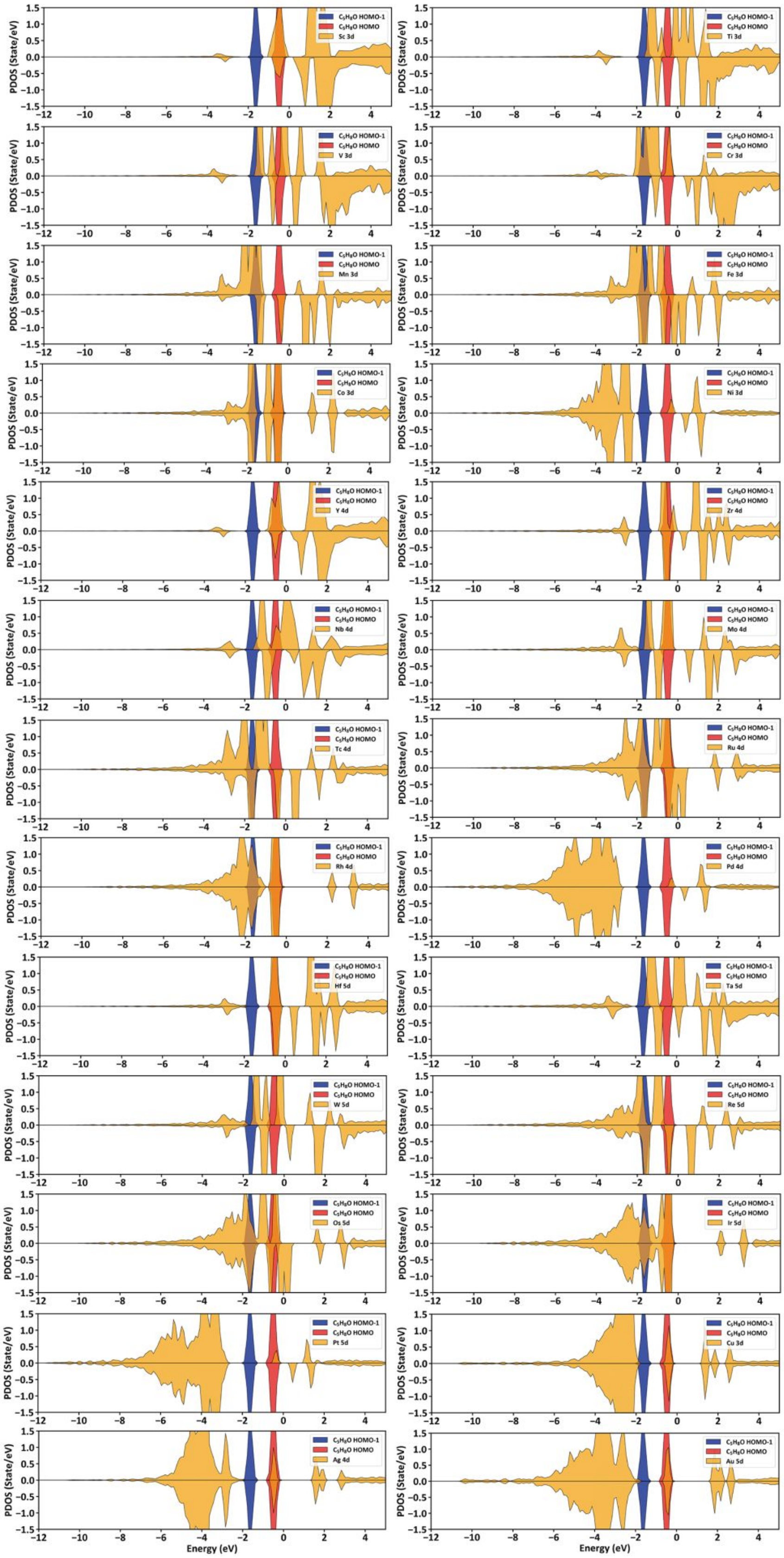

Figure S16. The overlap between the MOs (HOMO, red area, HOMO-1, blue area) of C_5H_8O and the PDOS of the *d*-states (yellow area) of M_1 on pristine M_1@*h*-BN.

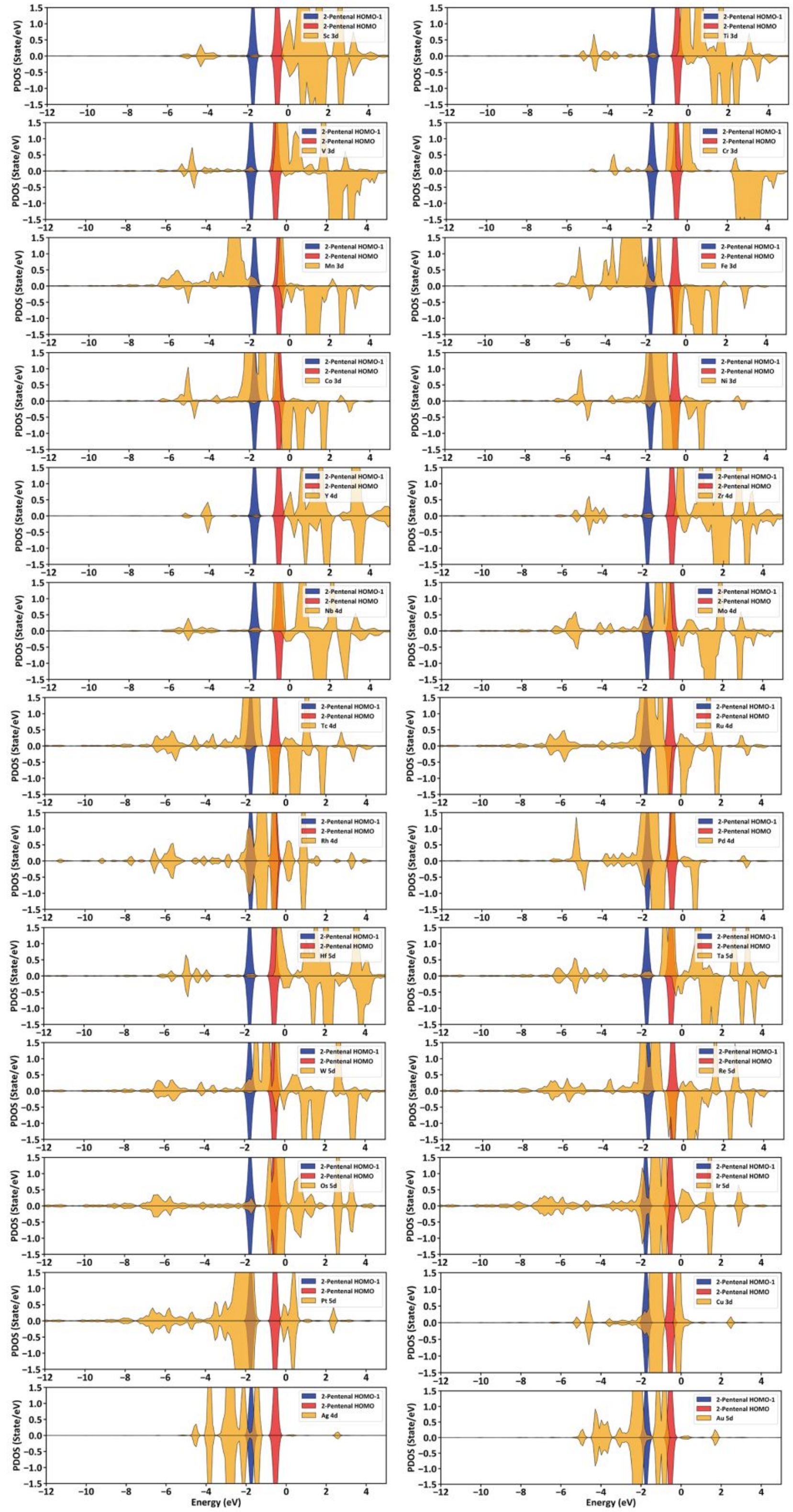

Figure S17. The overlap between the MOs (HOMO, red area, HOMO-1, blue area) of 2-pentenal and the PDOS of the *d*-states (yellow area) of M_1 on pristine $M_1@C_2N$.

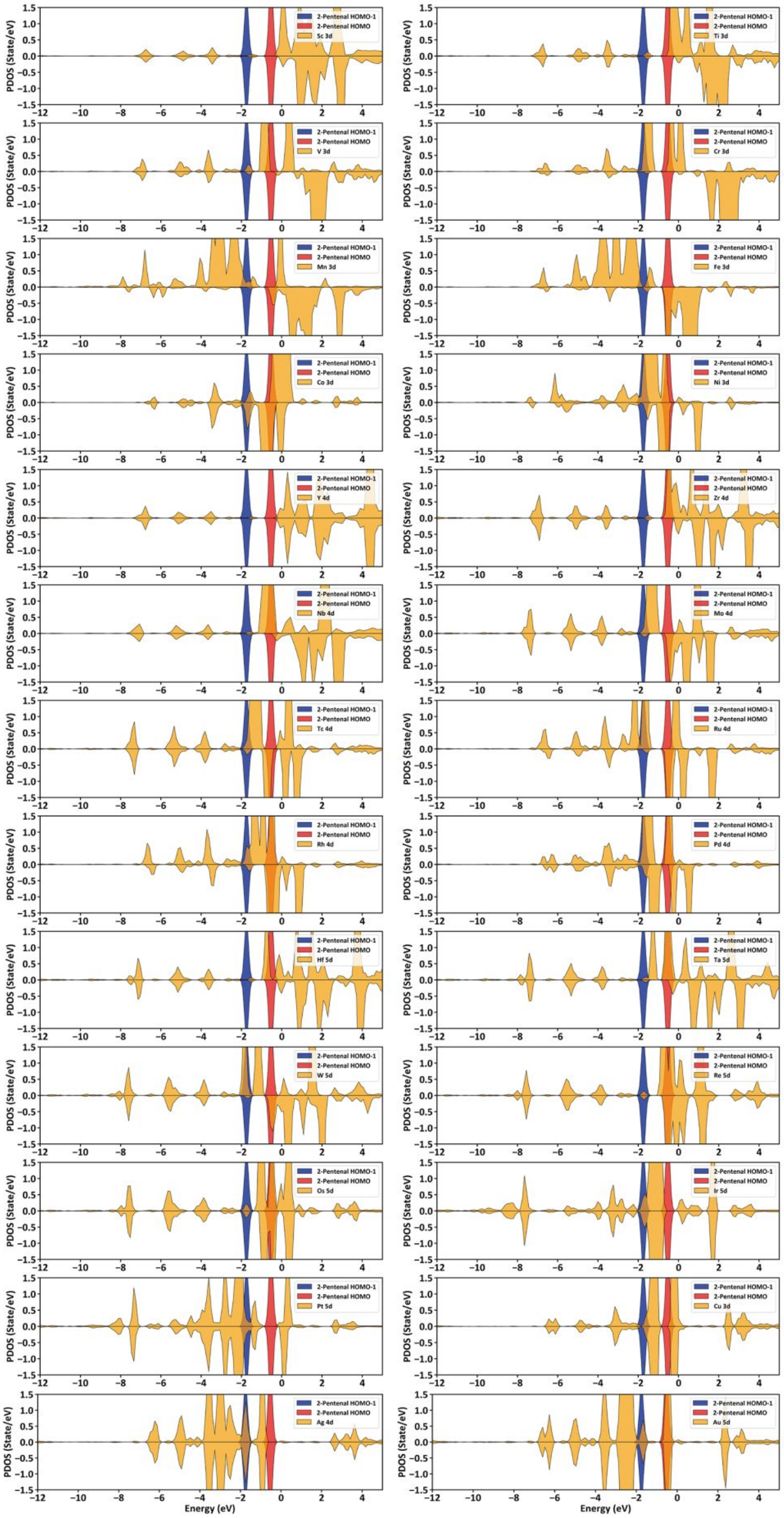

Figure S18. The overlap between the MOs (HOMO, red area, HOMO-1, blue area) of 2-pentenal and the PDOS of the *d*-states (yellow area) of M_1 on pristine M_1@*g*-C_3N_4.

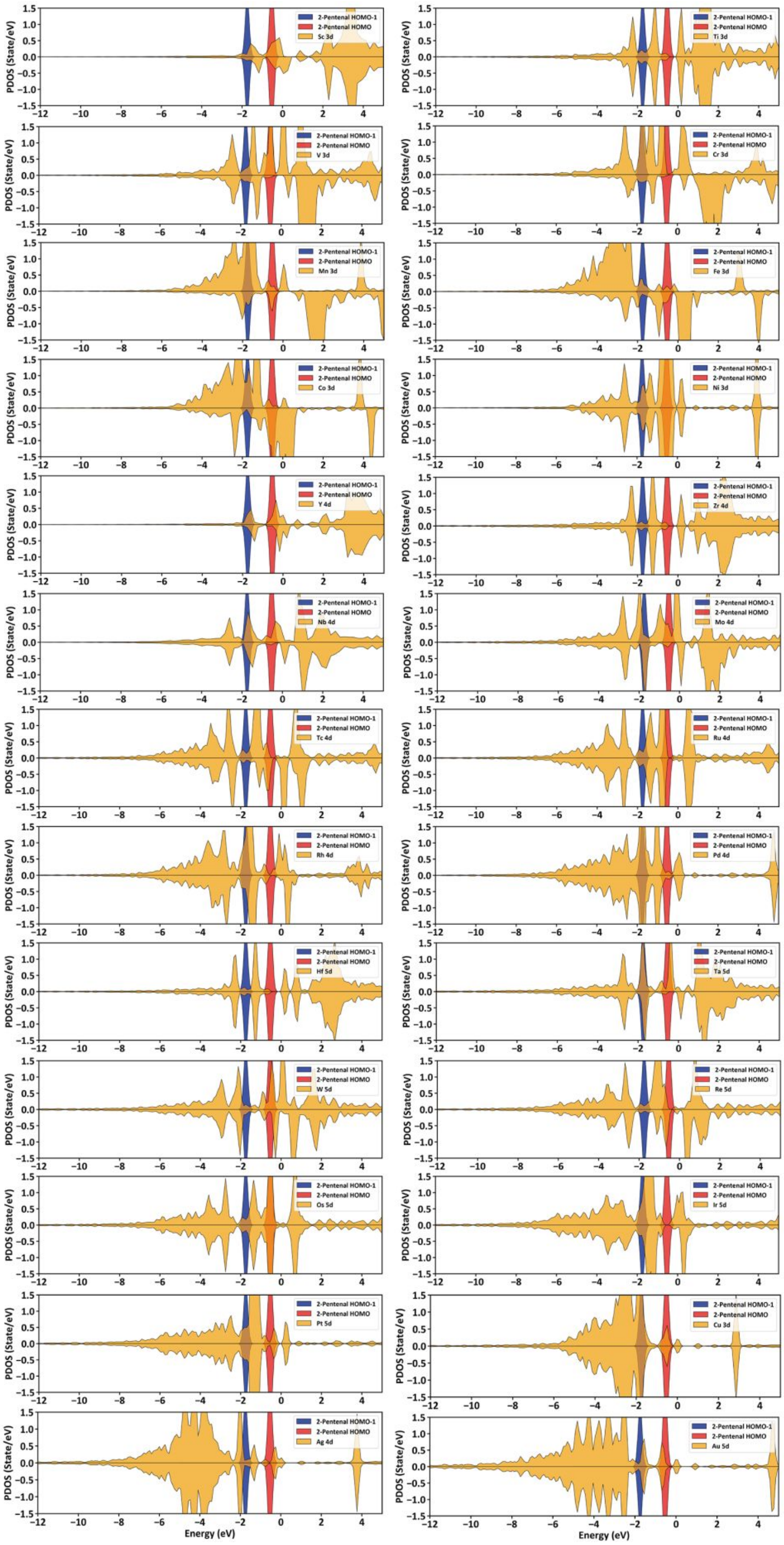

Figure S19. The overlap between the MOs (HOMO, red area, HOMO-1, blue area) of 2-pentenal and the PDOS of the *d*-states (yellow area) of M_1 on pristine M_1@DG.

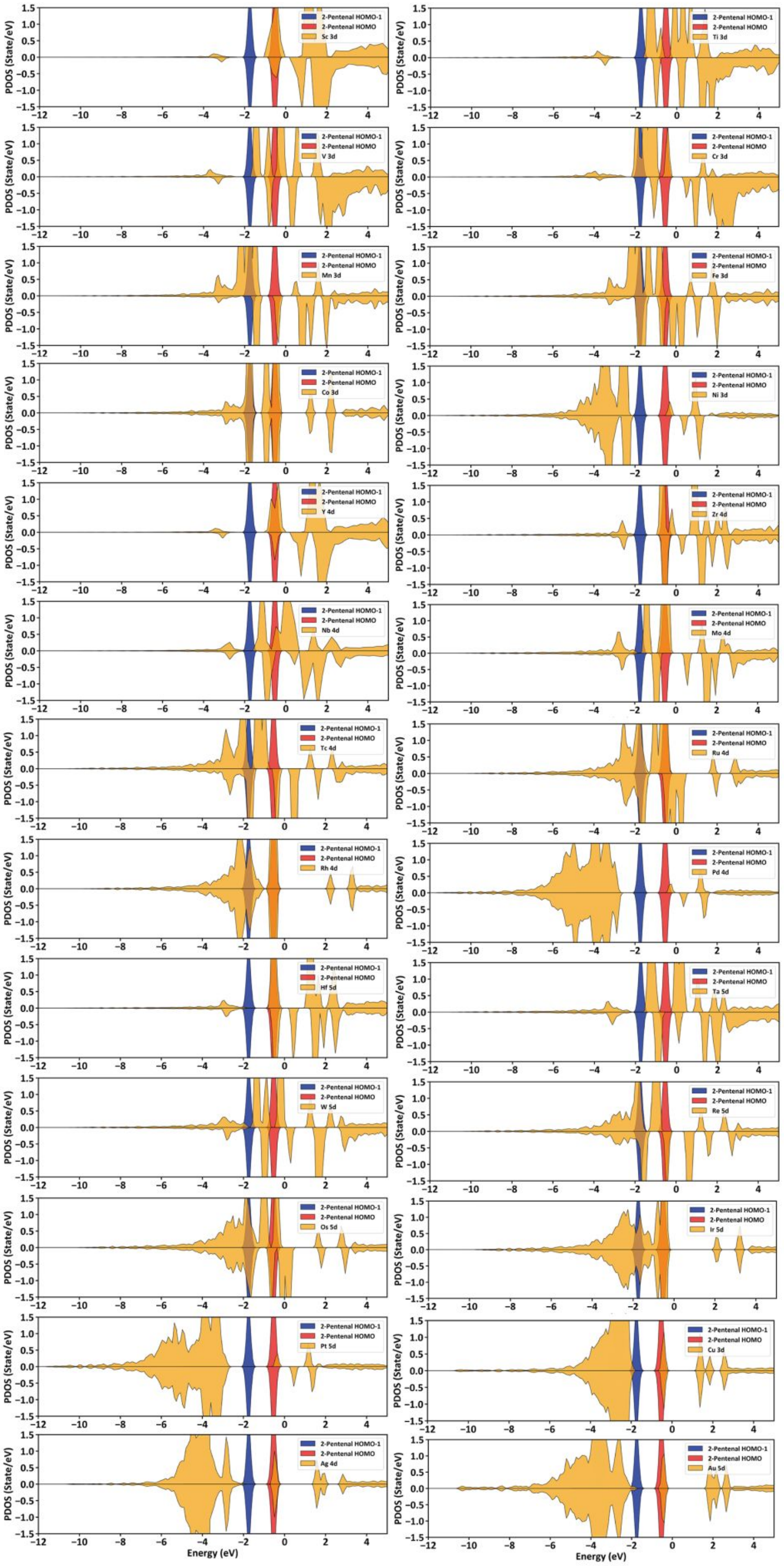

Figure S20. The overlap between the MOs (HOMO, red area, HOMO-1, blue area) of 2-pentenal and the PDOS of the *d*-states (yellow area) of M_1 on pristine M_1@*h*-BN.

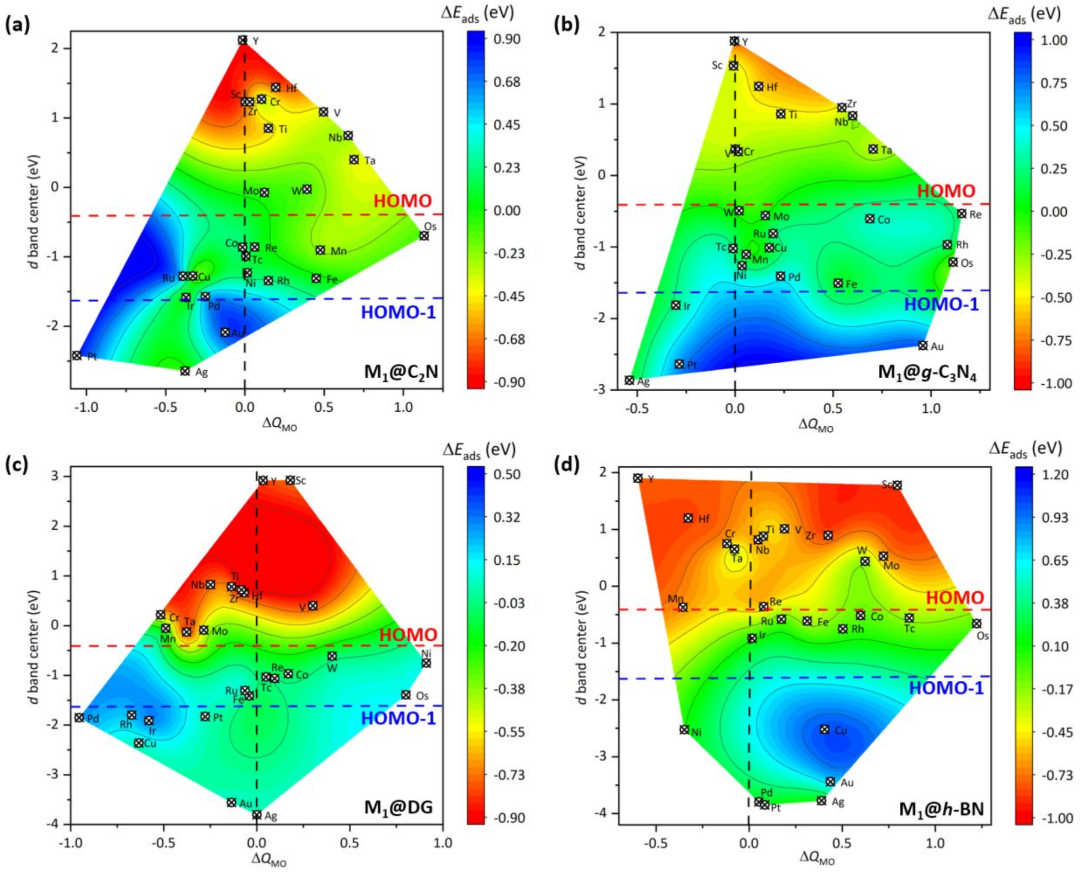

Figure S21. The 2D contour diagrams of the ΔE_{ads} of C_4H_6O as a functional of the *d*-band center of M_1 with the difference between the *d*-band and ΔQ_{MO} on (a) M_1@C_2N, (b) M_1@*g*-C_3N_4, (c) M_1@DG, and (d) M_1@*h*-BN.

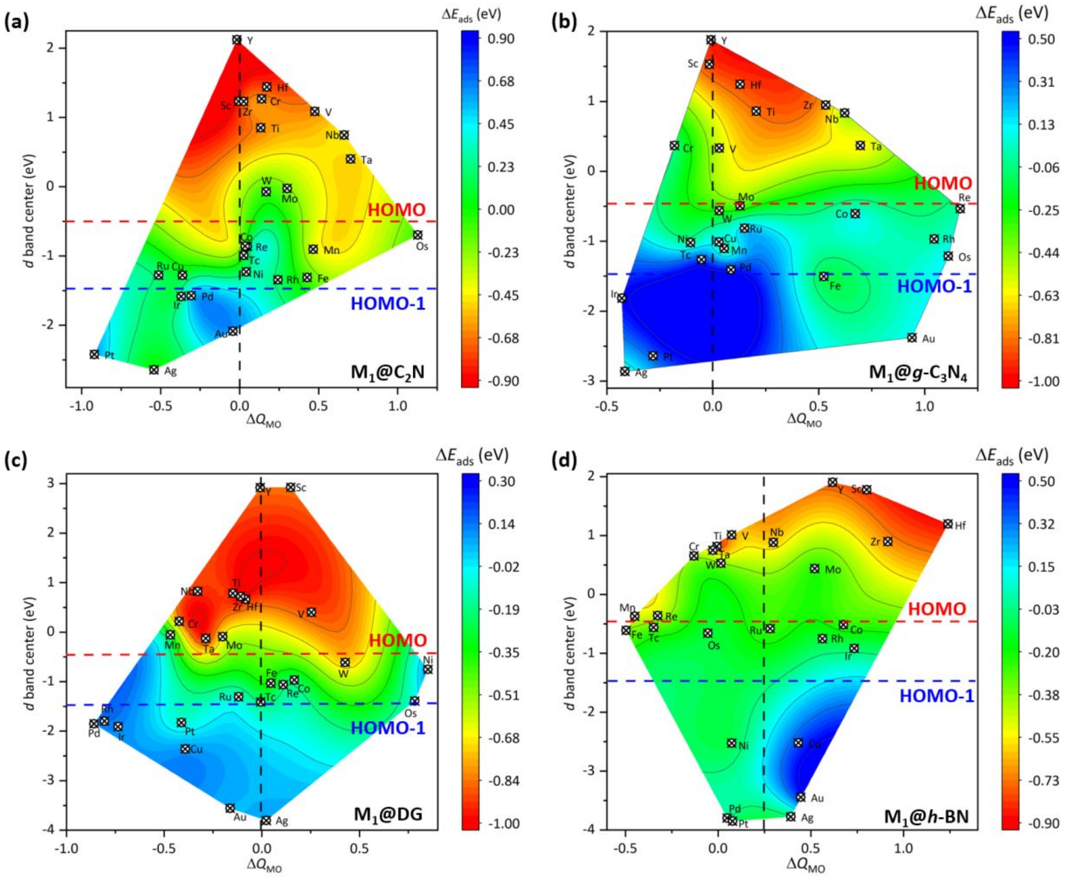

Figure S22. The 2D contour diagrams of the ΔE_{ads} of C_5H_8O as a functional of the *d*-band center of M_1 with the difference between the *d*-band and ΔQ_{MO} on (a) M_1@C_2N, (b) M_1@*g*-C_3N_4, (c) M_1@DG, and (d) M_1@*h*-BN.

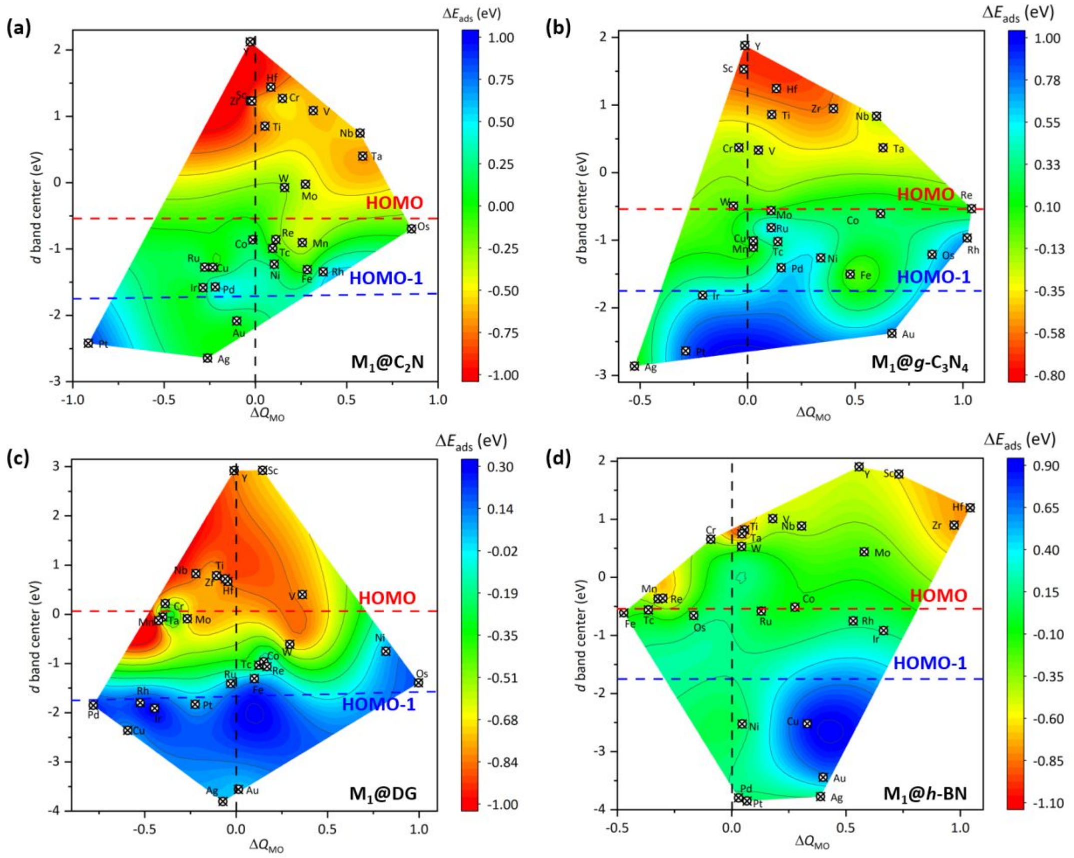

Figure S23. The 2D contour diagrams of the ΔE_{ads} of 2-pentenal as a functional of the *d*-band center of M_1 with the difference between the *d*-band and MO overlap (ΔQ_{MO}) on (a) M_1@C_2N, (b) M_1@*g*-C_3N_4, (c) M_1@DG, and (d) M_1@*h*-BN.

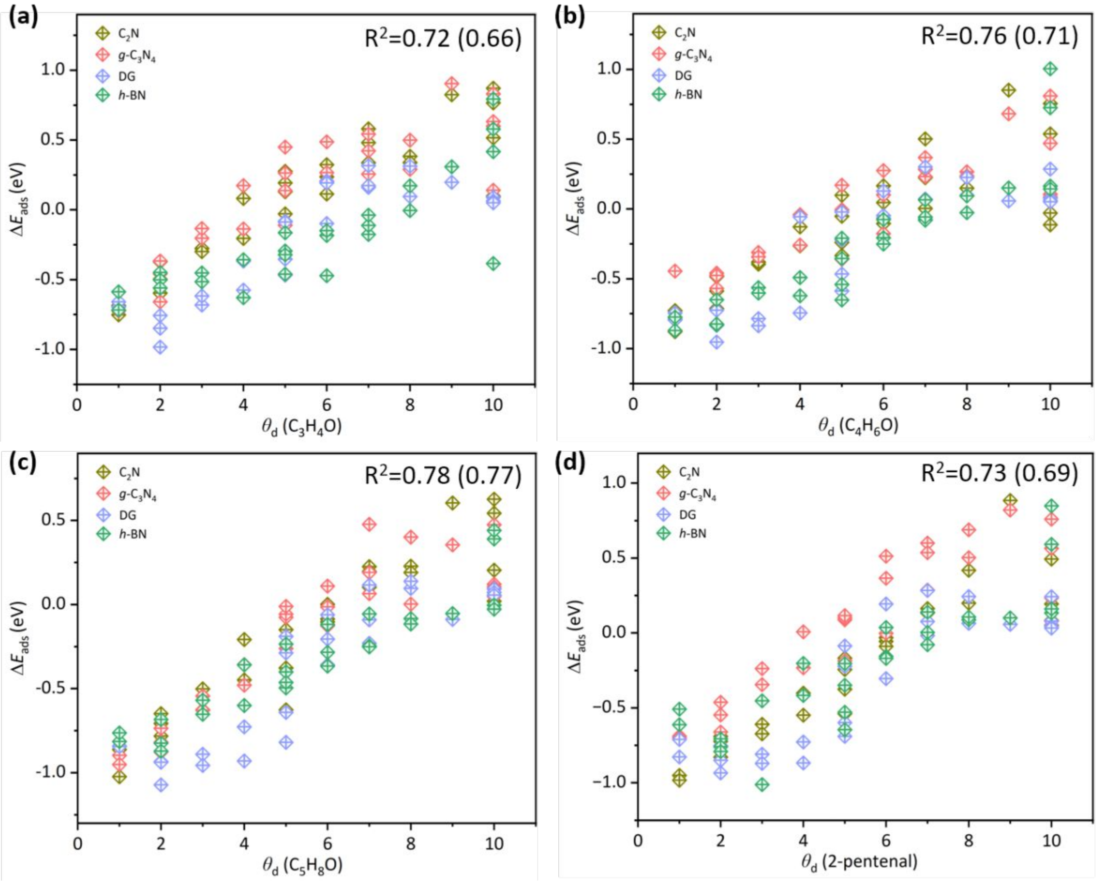

Figure S24. Adsorption energy differences (ΔE_{ads}) of (a) C_3H_4O, (b) C_4H_6O, (c) C_5H_8O and (d) 2-pentenal on the different SAC (M_1@C_2N, M_1@*g*-C_3N_4, M_1@DG and M_1@*h*-BN) as a function of θ_d of M_1. The R^2 for the IB (Cu, Ag, and Au) is shown in parentheses.

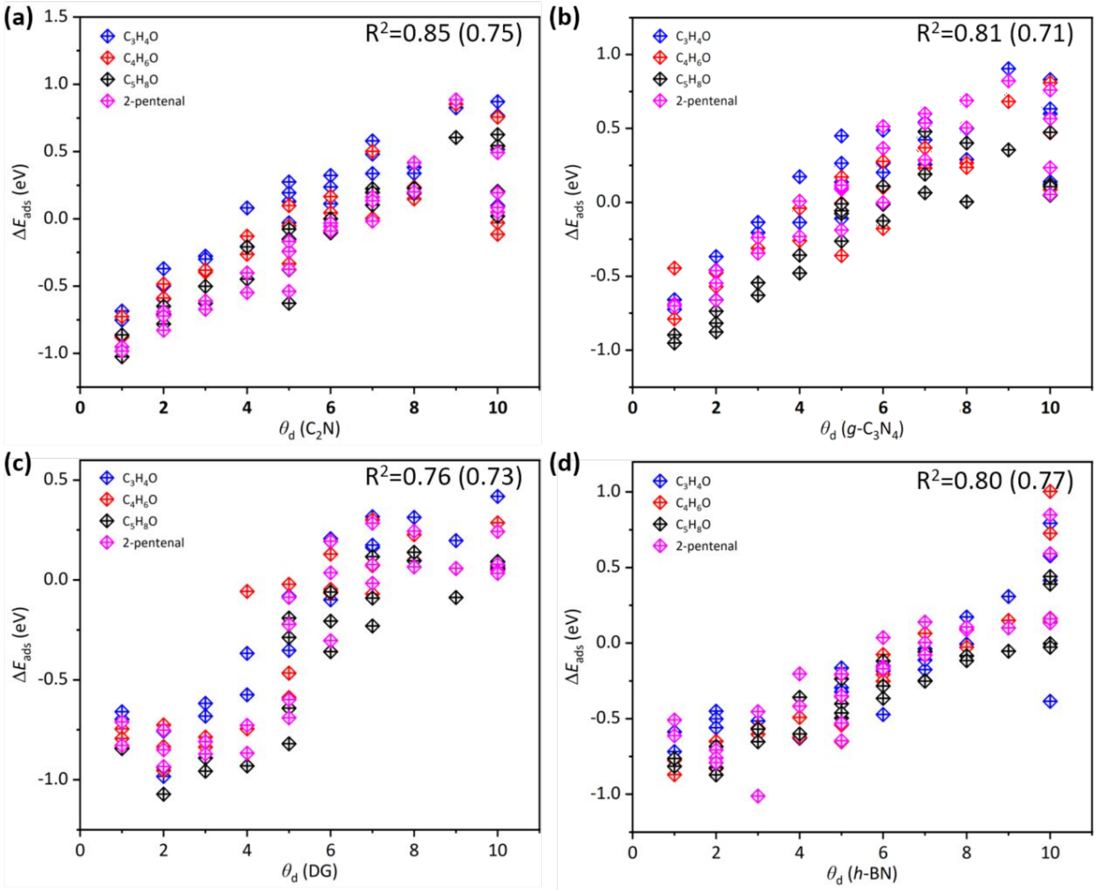

Figure S25. Adsorption energy differences (ΔE_{ads}) of C_3H_4O, C_4H_6O, C_5H_8O and 2-pentenal on the same SAC of (a) M_1@C_2N, (b) M_1@*g*-C_3N_4, (c) M_1@DG and (d) M_1@*h*-BN as a function of θ_d of M_1. The R^2 for the IB (Cu, Ag, and Au) is shown in parentheses.

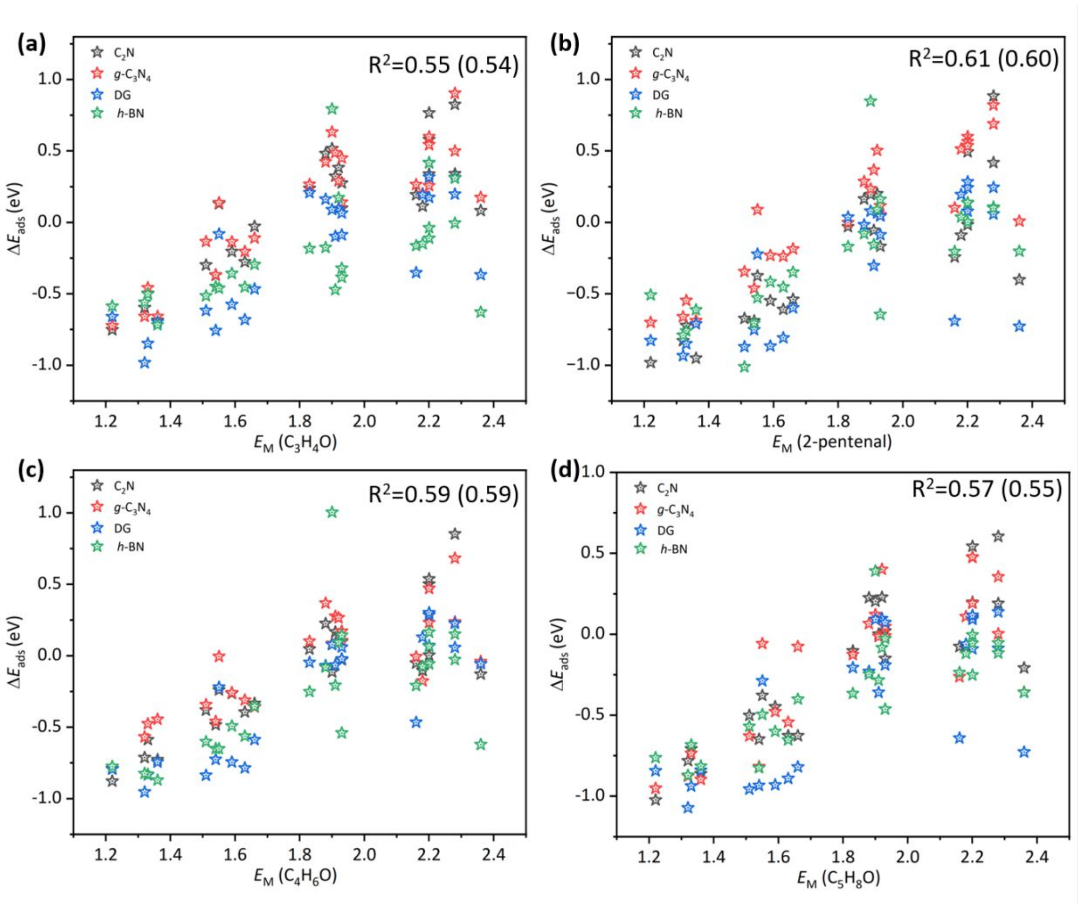

Figure S26. Adsorption energy differences (ΔE_{ads}) of (a) C_3H_4O, (b) C_4H_6O, (c) C_5H_8O and (d) 2-pentenal on the different SAC (M_1@C_2N, M_1@*g*-C_3N_4, M_1@DG and M_1@*h*-BN) as a function of E_M of M_1. The R^2 for the IB (Cu, Ag, and Au) is shown in parentheses.

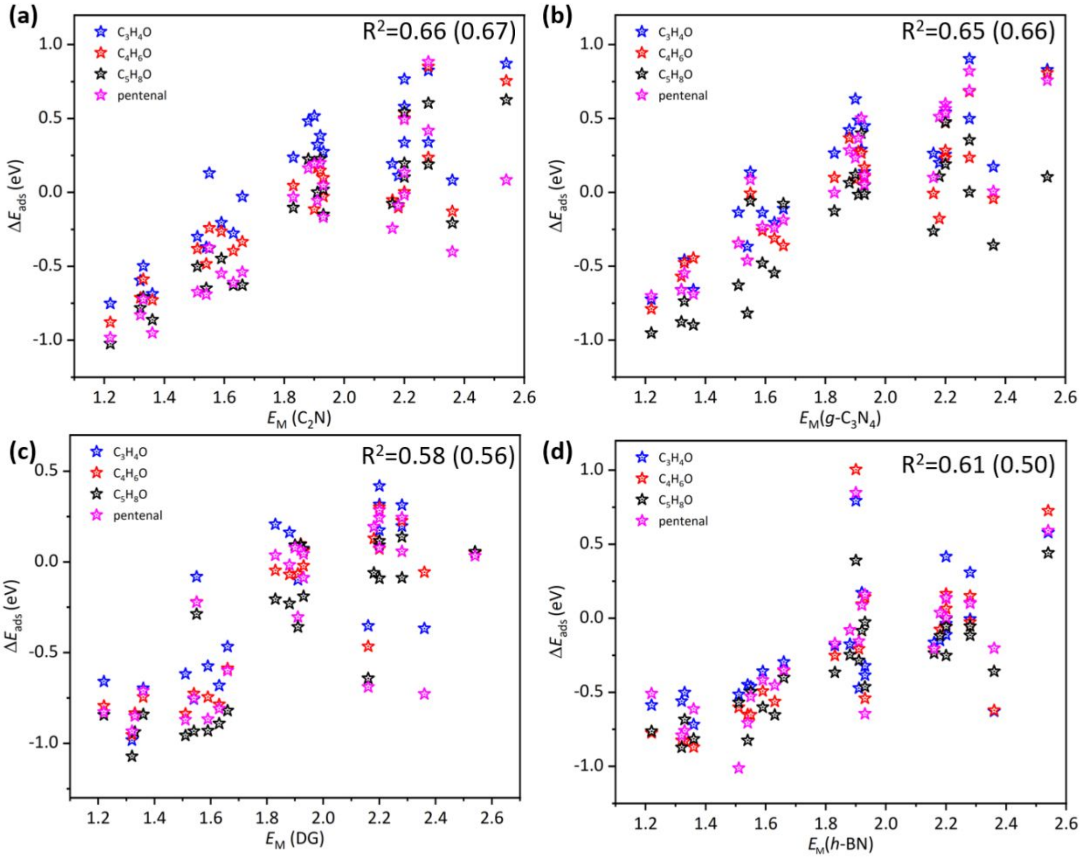

Figure S27. Adsorption energy differences (ΔE_{ads}) of C_3H_4O, C_4H_6O, C_5H_8O and 2-pentenal on the same SAC of (a) $M_1@C_2N$, (b) $M_1@g$-C_3N_4, (c) M_1@DG and (d) $M_1@h$-BN as a function of E_M of M_1. The R^2 for the IB (Cu, Ag, and Au) is shown in parentheses.

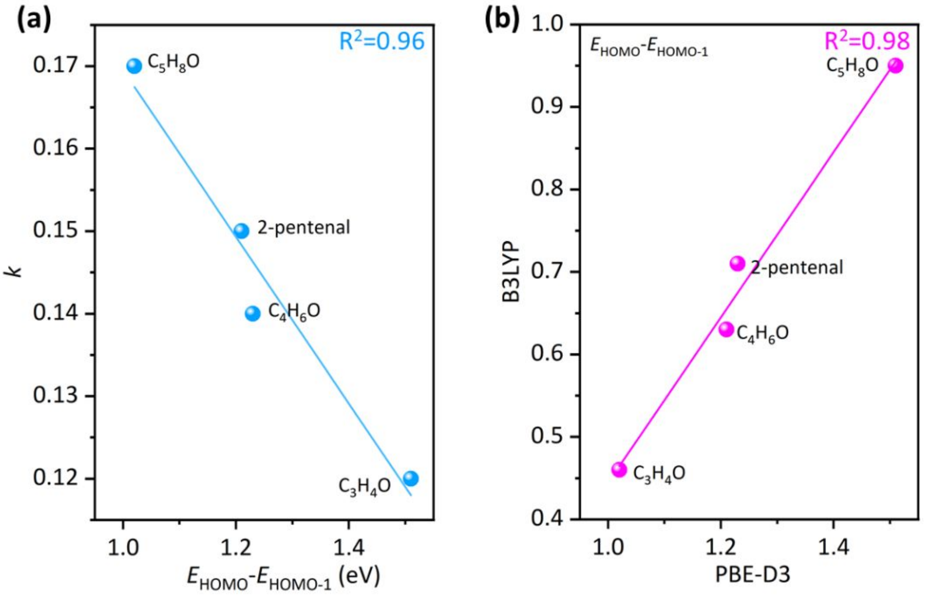

Figure S28. (a) The relationship between E_{HOMO}-E_{HOMO-1} and k. (b) The relationship between E_{HOMO}-$E_{(HOMO-1)}$ using PBE-D3 method and E_{HOMO}- $E_{(HOMO-1)}$ using B3LYP method.

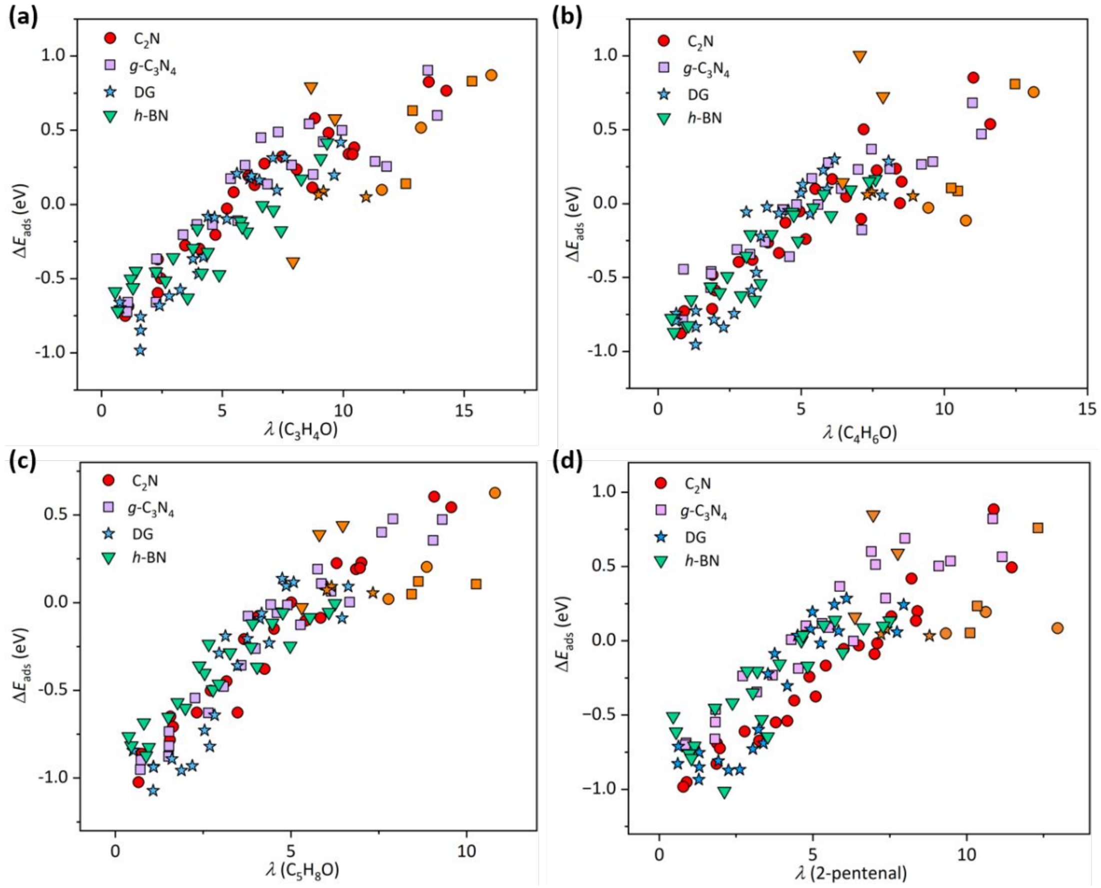

Figure S29. The ΔE_{ads} of the adsorbed (a) C_3H_4O, (b) C_4H_6O, (c) C_5H_8O and (d) 2-pentenal as a function of descriptor λ on M_1@C_2N, M_1@*g*-C_3N_4, M_1@DG, and M_1@*h*-BN with the IB (orange dots) considered.

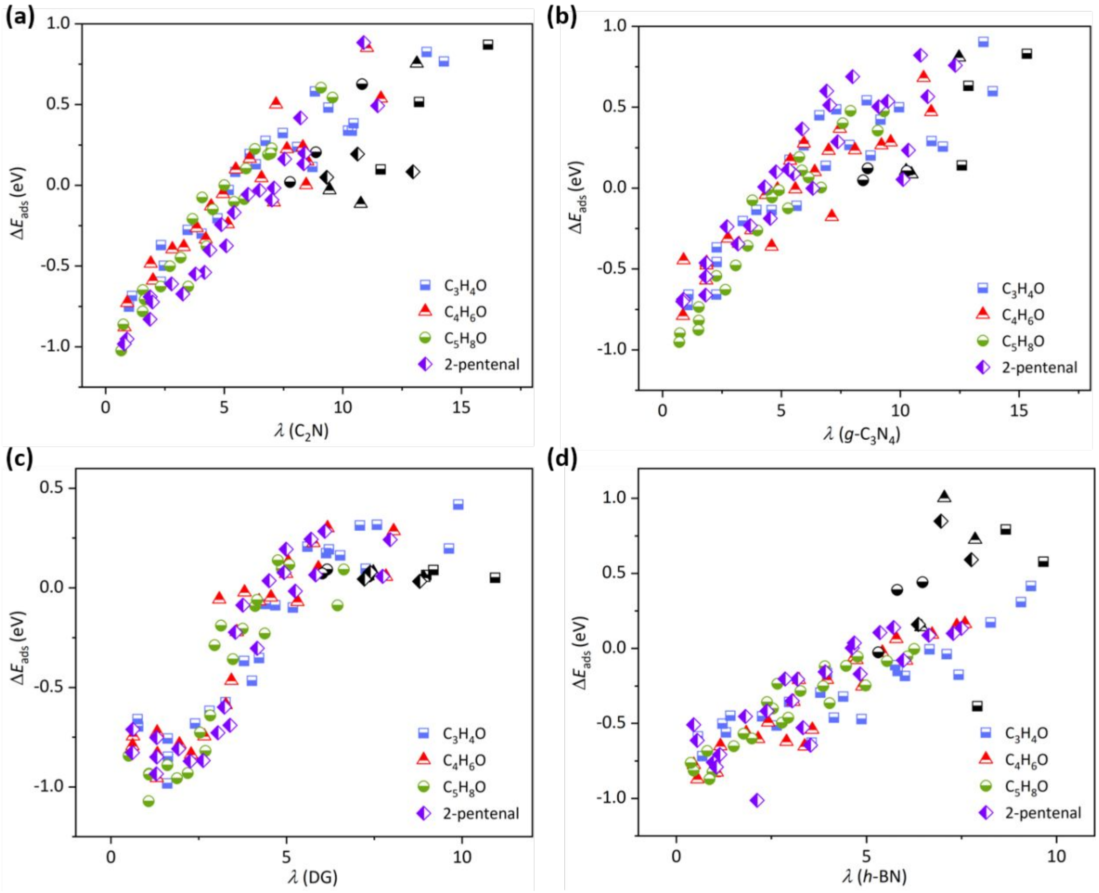

Figure S30. The ΔE_{ads} of the adsorbed C_3H_4O, C_4H_6O, C_5H_8O and 2-pentenal as a function of descriptor λ on (a) $M_1@C_2N$, (b) $M_1@g\text{-}C_3N_4$, (c) $M_1@DG$ and (d) $M_1@h\text{-}BN$ with the IB (black dots) considered.

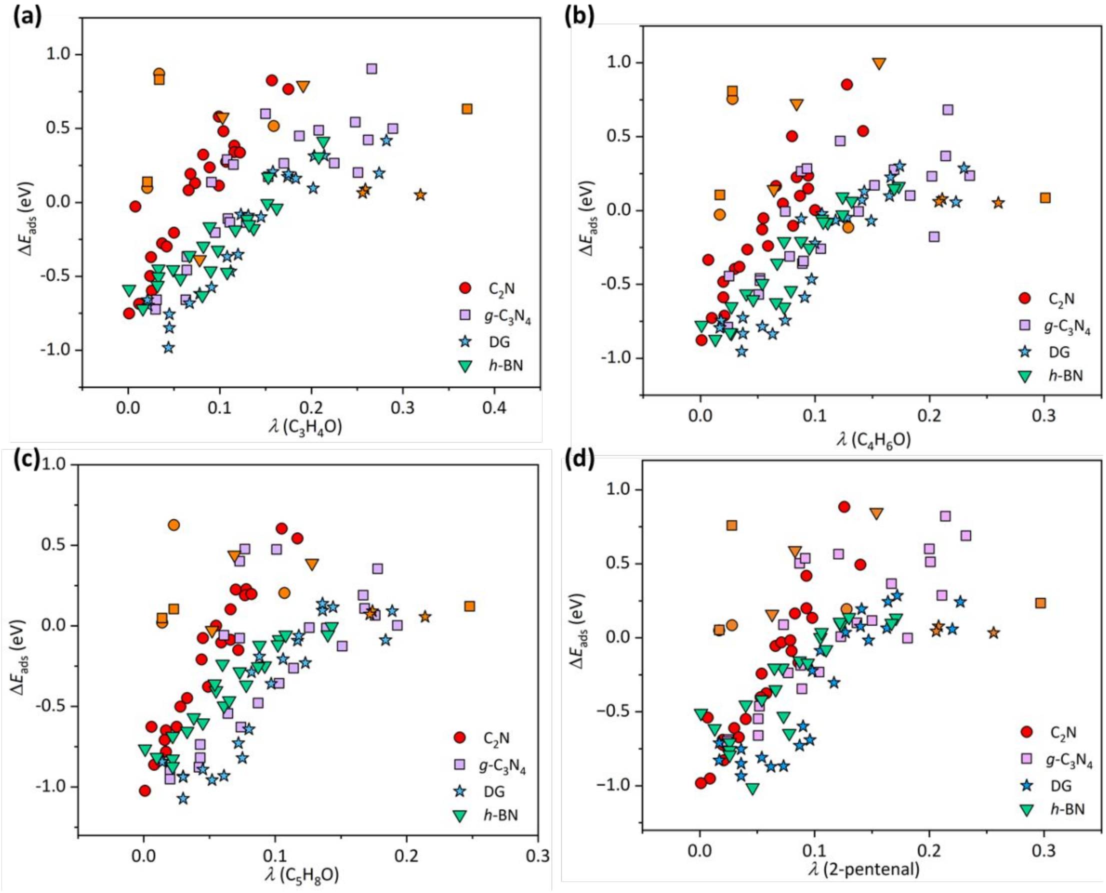

Figure S31. The ΔE_{ads} of same α, β-UAL (a) C_3H_4O, (b) C_4H_6O, (c) C_5H_8O and (d) 2-pentenal as a function of descriptor λ on four SACs without regard to outer CE. The data for IB is represented by orange dots.

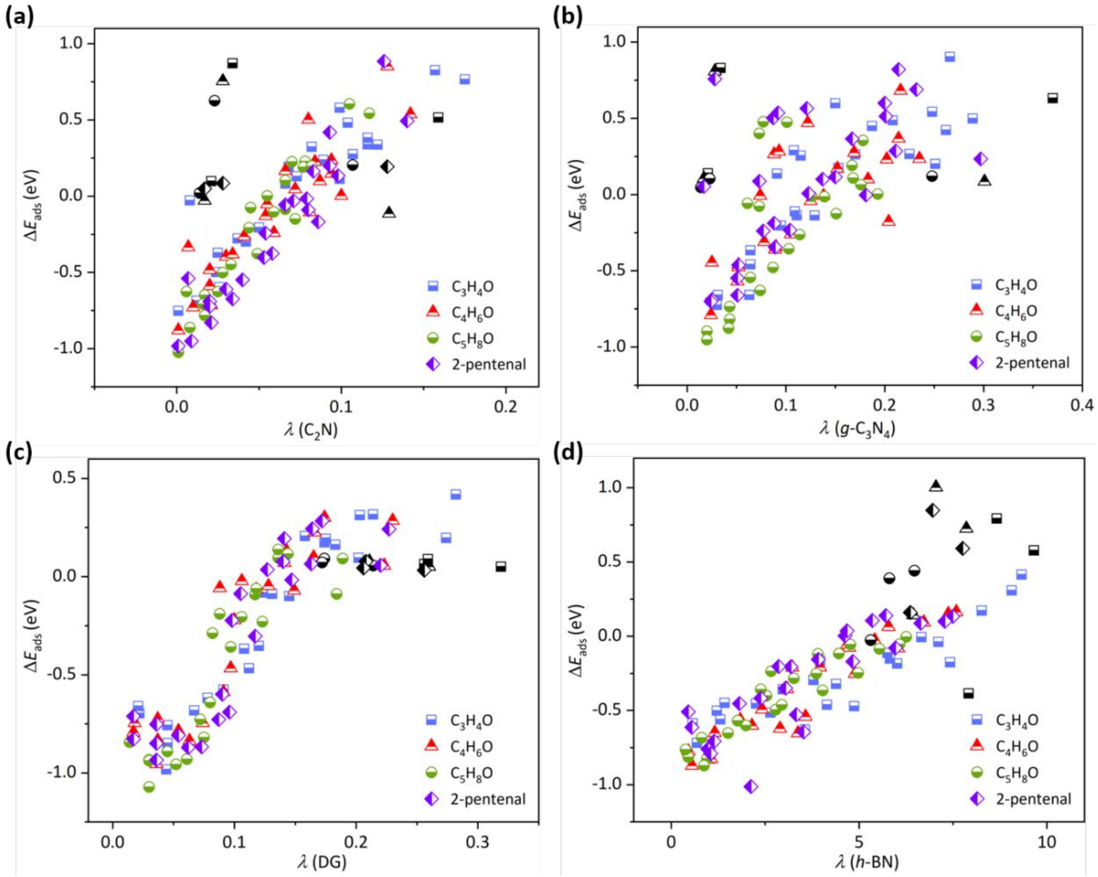

Figure S32. The ΔE_{ads} of each α, β-UAL as a function of descriptor λ on four SACs (a) $M_1@C_2N$, (b) g-C_3N_4, (c) M_1@DG and (d) $M_1@h$-BN without regard to outer CE. The data for IB is represented by black dots.

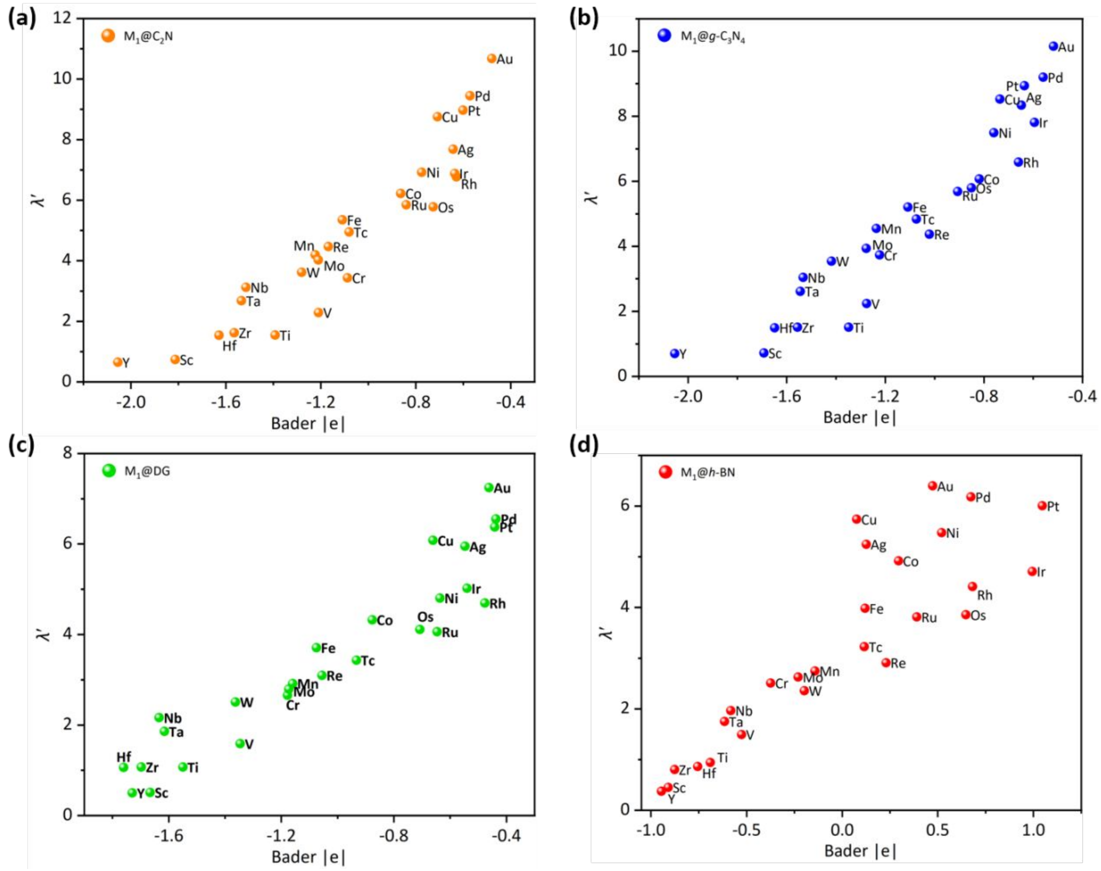

Figure S33. The Bader charges of M_1 versus λ' ($\theta_d \cdot IE \cdot \left[E_M + \sum_{i=1}^{n}\sum_{j=1}^{m+1}\frac{E_j}{2}\cdot\left(\cos\left(\frac{\pi \cdot r_{ij}}{r_c}\right)+1\right)\right]\cdot 10^{-6}$) on (a) M_1@C_2N, (b) M_1@*g*-C_3N_4, (c) M_1@*g*-DG and (d) M_1@*h*-BN

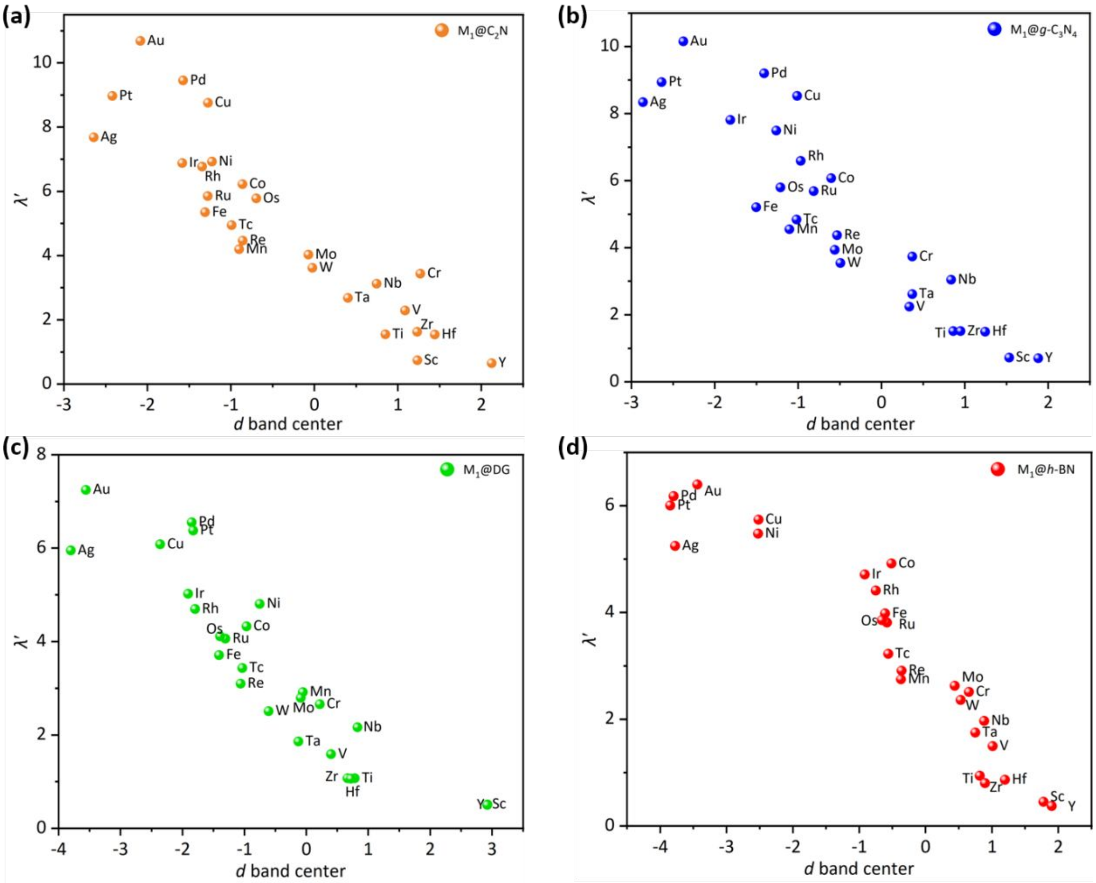

Figure S34. The d-band center of M_1 versus $\lambda'(\theta_d \cdot IE \cdot \left[E_M + \sum_{i=1}^{n}\sum_{j=1}^{m+1}\frac{E_j}{2}\cdot\left(\cos\left(\frac{\pi \cdot r_{ij}}{r_c}\right)+1\right)\right]\cdot 10^{-6})$ on (a) M_1@C_2N, (b) M_1@g-C_3N_4, (c) M_1@g-DG and (d) M_1@h-BN.

2. Supporting Tables

Table S1. Adsorption energies of *ald-mode* ($E_{\text{ald-mode}}$, eV) and *ene-mode* ($E_{\text{ene-mode}}$, eV), with their differences (ΔE_{ads}, eV) for C_3H_4O, C_4H_6O, C_5H_8O and 2-pentenal adsorbed on $M_1@C_2N$.

	C_3H_4O			C_4H_6O			C_5H_8O			2-pentenal		
$M_1@C_2N$	$E_{ald\text{-}mode}$	$E_{ene\text{-}mode}$	ΔE_{ads}	$E_{ald\text{-}mode}$	$E_{ene\text{-}mode}$	ΔE_{ads}	$E_{ald\text{-}mode}$	$E_{ene\text{-}}$	ΔE_{ads}	$E_{ald\text{-}mode}$	$E_{ene\text{-}mode}$	ΔE_{ads}
Sc	-2.04	-1.35	-0.69	-2.06	-1.34	-0.73	-2.23	-1.37	-0.86	-2.20	-1.25	-0.95
Ti	-2.05	-1.68	-0.37	-2.03	-1.55	-0.48	-2.19	-1.54	-0.65	-2.10	-1.41	-0.69
V	-1.46	-1.18	-0.28	-1.44	-1.04	-0.39	-1.59	-0.97	-0.63	-1.56	-0.95	-0.61
Cr	-1.36	-1.33	-0.03	-1.48	-1.15	-0.33	-1.86	-1.23	-0.63	-1.60	-1.07	-0.54
Mn	-2.86	-2.99	0.13	-3.16	-2.92	-0.24	-3.29	-2.91	-0.38	-1.31	-0.94	-0.37
Y	-2.17	-1.42	-0.75	-2.18	-1.30	-0.88	-2.36	-1.34	-1.02	-2.16	-1.18	-0.98
Zr	-2.54	-2.05	-0.50	-2.55	-1.96	-0.59	-2.66	-1.96	-0.71	-2.54	-1.81	-0.72
Nb	-2.15	-1.94	-0.21	-2.12	-1.85	-0.26	-2.24	-1.79	-0.45	-2.22	-1.67	-0.55
Mo	-1.52	-1.71	0.19	-1.55	-1.50	-0.05	-1.64	-1.57	-0.08	-1.67	-1.43	-0.24
Tc	-1.39	-1.71	0.32	-1.39	-1.56	0.17	-1.56	-1.56	0.00	-1.47	-1.42	-0.06
Fe	-1.10	-1.33	0.24	-1.12	-1.17	0.05	-1.30	-1.19	-0.10	-1.14	-1.11	-0.03
Co	-1.00	-1.48	0.48	-1.04	-1.26	0.23	-1.13	-1.35	0.23	-1.11	-1.27	0.16
Ni	-0.90	-1.28	0.38	-0.81	-0.96	0.15	-0.97	-1.20	0.23	-0.87	-1.07	0.20
Ru	-1.02	-1.60	0.58	-1.02	-1.52	0.50	-1.62	-1.72	0.10	-1.38	-1.36	-0.02
Rh	-1.15	-1.49	0.34	-1.08	-1.32	0.24	-1.31	-1.50	0.19	-0.87	-1.28	0.42
Pd	-0.52	-1.29	0.77	-0.61	-1.15	0.54	-0.75	-1.29	0.54	-0.64	-1.14	0.49
Os	-1.80	-1.92	0.11	-1.82	-1.71	-0.10	-1.91	-1.82	-0.09	-1.64	-1.55	-0.09
Ir	-0.85	-1.18	0.34	-0.88	-0.89	0.00	-0.96	-1.16	0.20	-0.77	-0.90	0.13
Pt	-1.02	-1.84	0.83	-0.33	-1.18	0.85	-1.04	-1.64	0.60	-0.81	-1.69	0.88
Hf	-2.73	-2.13	-0.60	-2.75	-2.04	-0.71	-2.83	-2.05	-0.78	-2.73	-1.90	-0.83
Ta	-2.48	-2.18	-0.30	-2.52	-2.14	-0.38	-2.59	-2.09	-0.50	-2.62	-1.95	-0.67
W	-2.10	-2.18	0.08	-2.05	-1.92	-0.13	-2.22	-2.01	-0.21	-2.28	-1.88	-0.40
Re	-1.89	-2.16	0.27	-1.88	-1.97	0.10	-2.09	-1.94	-0.15	-1.99	-1.82	-0.17
Cu	-0.81	-1.32	0.52	-1.04	-0.92	-0.11	-0.99	-1.19	0.20	-0.87	-1.06	0.19
Ag	-0.38	-0.47	0.10	-0.45	-0.42	-0.03	-0.60	-0.62	0.02	-0.40	-0.45	0.05
Au	-0.30	-1.17	0.87	-0.37	-1.13	0.76	-0.45	-1.08	0.63	-0.39	-0.47	0.08

Table S2. Adsorption energies of *ald-mode* ($E_{\text{ald-mode}}$, eV) and *ene-mode* ($E_{\text{ene-mode}}$, eV), with their differences (ΔE_{ads}, eV) for C_3H_4O, C_4H_6O, C_5H_8O and 2-pentenal adsorbed on $M_1@g$-C_3N_4.

	C_3H_4O			C_4H_6O			C_5H_8O			2-pentenal		
$M_1@g$-C_3N_4	$E_{ald\text{-}mode}$	$E_{ene\text{-}mode}$	ΔE_{ads}	$E_{ald\text{-}mode}$	$E_{ene\text{-}mode}$	ΔE_{ads}	$E_{ald\text{-}mode}$	$E_{ene\text{-}mode}$	ΔE_{ads}	$E_{ald\text{-}mode}$	$E_{ene\text{-}mode}$	ΔE_{ads}
Sc	-3.38	-2.72	-0.66	-3.07	-2.62	-0.45	-3.51	-2.61	-0.90	-3.29	-2.60	-0.69
Ti	-3.07	-2.70	-0.37	-3.04	-2.58	-0.46	-3.21	-2.39	-0.82	-3.00	-2.54	-0.46
V	-2.50	-2.30	-0.20	-2.47	-2.16	-0.31	-2.63	-2.09	-0.54	-2.37	-2.13	-0.24
Cr	-2.46	-2.35	-0.11	-2.55	-2.19	-0.36	-2.30	-2.22	-0.08	-2.35	-2.16	-0.19
Mn	-4.55	-4.69	0.14	-4.56	-4.55	-0.01	-4.55	-4.49	-0.06	-4.55	-4.63	0.09
Y	-3.49	-2.77	-0.72	-3.53	-2.74	-0.79	-3.65	-2.70	-0.95	-3.38	-2.68	-0.70
Zr	-3.62	-3.16	-0.46	-3.45	-2.97	-0.48	-3.62	-2.88	-0.74	-3.48	-2.93	-0.55
Nb	-3.02	-2.88	-0.14	-3.02	-2.76	-0.26	-3.05	-2.57	-0.48	-2.97	-2.73	-0.23
Mo	-2.54	-2.80	0.26	-2.61	-2.60	-0.01	-2.73	-2.73	-0.26	-2.55	-2.65	0.10
Tc	-2.55	-3.04	0.49	-2.52	-2.80	0.28	-2.92	-2.91	-0.01	-2.56	-2.93	0.37
Fe	-2.70	-2.97	0.27	-2.76	-2.86	0.10	-2.82	-2.69	-0.13	-2.74	-2.74	0.00
Co	-2.73	-3.16	0.42	-2.86	-3.22	0.37	-2.92	-2.99	0.06	-2.72	-3.01	0.29
Ni	-3.01	-3.30	0.29	-2.78	-3.05	0.27	-2.72	-3.12	0.40	-2.70	-3.20	0.50
Ru	-2.57	-3.12	0.54	-2.95	-3.19	0.23	-2.68	-2.88	0.19	-2.67	-3.27	0.60
Rh	-3.66	-4.16	0.50	-2.55	-2.78	0.24	-2.72	-2.72	0.00	-2.17	-2.86	0.69
Pd	-3.05	-3.65	0.60	-2.77	-3.24	0.47	-2.70	-3.17	0.47	-2.87	-3.44	0.57
Os	-3.32	-3.52	0.20	-3.44	-3.27	-0.18	-3.17	-3.28	0.11	-2.61	-3.12	0.51
Ir	-2.40	-2.66	0.26	-2.44	-2.72	0.28	-2.52	-2.99	0.48	-2.16	-2.69	0.54
Pt	-3.45	-4.35	0.90	-3.19	-3.87	0.68	-3.57	-3.93	0.36	-3.44	-4.26	0.82
Hf	-3.82	-3.16	-0.66	-3.62	-3.05	-0.57	-3.83	-2.95	-0.88	-3.65	-2.99	-0.66
Ta	-3.42	-3.29	-0.13	-3.40	-3.05	-0.34	-3.49	-2.86	-0.63	-3.35	-3.00	-0.34
W	-2.91	-3.09	0.17	-3.02	-2.98	-0.04	-3.10	-2.74	-0.36	-2.92	-2.93	0.01
Re	-2.67	-3.12	0.45	-2.77	-2.94	0.17	-2.90	-2.89	-0.01	-2.55	-2.66	0.12
Cu	-2.39	-3.02	0.63	-2.71	-2.79	0.09	-2.91	-3.03	0.12	-2.61	-2.85	0.23
Ag	-2.28	-2.42	0.14	-2.29	-2.40	0.11	-2.42	-2.46	0.05	-2.32	-2.37	0.05
Au	-2.68	-3.51	0.83	-2.50	-3.31	0.81	-2.59	-2.69	0.10	-2.62	-3.38	0.76

Table S3. Adsorption energies of *ald-mode* ($E_{\text{ald-mode}}$, eV) and *ene-mode* ($E_{\text{ene-mode}}$, eV), with their differences (ΔE_{ads}, eV) for C_3H_4O, C_4H_6O, C_5H_8O and 2-pentenal adsorbed on M_1@DG.

	C_3H_4O			C_4H_6O			C_5H_8O			2-pentenal		
M_1@DG	$E_{ald\text{-}mode}$	$E_{ene\text{-}mode}$	ΔE_{ads}	$E_{ald\text{-}mode}$	$E_{ene\text{-}mode}$	ΔE_{ads}	$E_{ald\text{-}mode}$	$E_{ene\text{-}mode}$	ΔE_{ads}	$E_{ald\text{-}mode}$	$E_{ene\text{-}mode}$	ΔE_{ads}
Sc	-1.34	-0.64	-0.70	-1.47	-0.72	-0.75	-1.63	-0.79	-0.84	-1.45	-0.74	-0.71
Ti	-1.76	-1.01	-0.76	-1.83	-1.11	-0.73	-2.06	-1.12	-0.93	-1.87	-1.12	-0.75
V	-1.85	-1.17	-0.68	-1.95	-1.16	-0.79	-2.13	-1.23	-0.89	-1.99	-1.18	-0.81
Cr	-1.44	-0.97	-0.47	-1.56	-0.97	-0.59	-1.75	-0.93	-0.82	-1.60	-1.00	-0.60
Mn	-0.95	-0.87	-0.08	-1.08	-0.86	-0.22	-1.17	-0.89	-0.29	-1.05	-0.82	-0.22
Y	-1.17	-0.51	-0.66	-1.35	-0.55	-0.79	-1.45	-0.60	-0.84	-1.39	-0.56	-0.83
Zr	-1.75	-0.90	-0.85	-1.80	-0.97	-0.83	-1.96	-1.02	-0.94	-1.82	-0.97	-0.85
Nb	-2.01	-1.44	-0.58	-2.11	-1.37	-0.74	-2.31	-1.38	-0.93	-2.15	-1.29	-0.87
Mo	-1.92	-1.56	-0.35	-1.99	-1.52	-0.47	-2.14	-1.49	-0.64	-2.02	-1.33	-0.69
Tc	-1.45	-1.36	-0.10	-1.32	-1.25	-0.07	-1.66	-1.30	-0.36	-1.57	-1.27	-0.30
Fe	-0.67	-0.88	0.21	-0.88	-0.83	-0.05	-1.06	-0.86	-0.21	-0.83	-0.87	0.04
Co	-0.98	-1.14	0.16	-1.00	-0.93	-0.07	-1.12	-0.89	-0.23	-1.01	-1.00	-0.02
Ni	-0.26	-0.36	0.10	-0.36	-0.46	0.10	-0.46	-0.55	0.10	-0.37	-0.44	0.07
Ru	-0.95	-1.13	0.17	-0.99	-1.07	0.07	-1.17	-1.08	-0.09	-1.01	-1.09	0.08
Rh	-0.93	-1.24	0.31	-0.95	-1.18	0.23	-1.08	-1.22	0.14	-0.96	-1.20	0.24
Pd	-0.27	-0.69	0.42	-0.34	-0.63	0.29	-0.45	-0.54	0.09	-0.43	-0.67	0.24
Os	-1.31	-1.50	0.19	-1.28	-1.41	0.13	-1.49	-1.43	-0.06	-1.25	-1.44	0.19
Ir	-1.16	-1.47	0.32	-1.10	-1.40	0.30	-1.29	-1.40	0.12	-1.13	-1.41	0.28
Pt	-0.28	-0.48	0.20	-0.35	-0.40	0.06	-0.48	-0.39	-0.09	-0.38	-0.44	0.06
Hf	-2.06	-1.07	-0.98	-2.09	-1.14	-0.95	-2.25	-1.18	-1.07	-2.08	-1.15	-0.93
Ta	-2.40	-1.78	-0.62	-2.50	-1.66	-0.84	-2.63	-1.67	-0.96	-2.55	-1.68	-0.87
W	-2.40	-2.03	-0.37	-2.20	-2.14	-0.06	-2.62	-1.89	-0.73	-2.49	-1.76	-0.73
Re	-1.84	-1.75	-0.09	-1.66	-1.63	-0.02	-1.85	-1.66	-0.19	-1.69	-1.60	-0.09
Cu	-0.28	-0.37	0.09	-0.38	-0.45	0.08	-0.48	-0.58	0.09	-0.39	-0.47	0.08
Ag	-0.28	-0.35	0.06	-0.37	-0.43	0.06	-0.48	-0.55	0.07	-0.38	-0.43	0.04
Au	-0.29	-0.34	0.05	-0.37	-0.42	0.05	-0.50	-0.55	0.06	-0.38	-0.42	0.03

Table S4. Adsorption energies of *ald-mode* ($E_{\text{ald-mode}}$, eV) and *ene-mode* ($E_{\text{ene-mode}}$, eV), with their differences (ΔE_{ads}, eV) for C_3H_4O, C_4H_6O, C_5H_8O and 2-pentenal adsorbed on M_1@*h*-BN (N_v).

	C_3H_4O			C_4H_6O			C_5H_8O			2-pentenal		
M_1@*h*-BN	$E_{ald\text{-}mode}$	$E_{ene\text{-}mode}$	ΔE_{ads}	$E_{ald\text{-}mode}$	$E_{ene\text{-}mode}$	ΔE_{ads}	$E_{ald\text{-}mode}$	$E_{ene\text{-}mode}$	ΔE_{ads}	$E_{ald\text{-}mode}$	$E_{ene\text{-}mode}$	ΔE_{ads}
Sc	-2.55	-1.83	-0.72	-2.47	-1.60	-0.87	-2.58	-1.77	-0.81	-1.78	-1.16	-0.61
Ti	-2.56	-2.11	-0.45	-2.54	-1.89	-0.65	-2.54	-1.72	-0.82	-2.61	-1.90	-0.71
V	-2.38	-1.92	-0.45	-2.36	-1.80	-0.56	-2.43	-1.77	-0.65	-2.28	-1.83	-0.45
Cr	-2.31	-2.02	-0.30	-2.29	-1.93	-0.36	-2.35	-1.94	-0.40	-2.30	-1.95	-0.35
Mn	-2.19	-1.73	-0.46	-2.26	-1.61	-0.65	-2.25	-1.75	-0.50	-2.19	-1.66	-0.53
Y	-2.47	-1.88	-0.59	-2.56	-1.79	-0.77	-2.79	-2.03	-0.76	-2.35	-1.84	-0.51
Zr	-1.93	-1.43	-0.50	-2.41	-1.58	-0.83	-1.91	-1.23	-0.68	-2.43	-1.67	-0.76
Nb	-1.97	-1.62	-0.36	-1.96	-1.47	-0.49	-2.03	-1.43	-0.60	-1.91	-1.49	-0.42
Mo	-1.53	-1.36	-0.16	-1.53	-1.32	-0.21	-1.56	-1.33	-0.24	-1.52	-1.32	-0.20
Tc	-1.92	-1.45	-0.47	-1.90	-1.70	-0.21	-1.83	-1.54	-0.28	-1.87	-1.72	-0.16
Fe	-2.27	-2.09	-0.18	-2.28	-2.02	-0.25	-2.32	-1.95	-0.37	-2.24	-2.07	-0.17
Co	-1.85	-1.67	-0.18	-1.85	-1.76	-0.08	-1.92	-1.68	-0.25	-1.85	-1.77	-0.08
Ni	-1.69	-1.86	0.17	-1.64	-1.73	0.09	-1.81	-1.73	-0.08	-1.65	-1.74	0.09
Ru	-1.60	-1.49	-0.11	-1.61	-1.55	-0.06	-1.66	-1.41	-0.25	-1.56	-1.57	0.00
Rh	-1.06	-1.05	-0.01	-1.16	-1.13	-0.03	-1.21	-1.10	-0.12	-1.10	-1.20	0.11
Pd	-1.01	-1.42	0.42	-1.01	-1.17	0.17	-1.18	-1.17	0.00	-1.03	-1.17	0.13
Os	-1.92	-1.77	-0.15	-1.93	-1.85	-0.08	-1.80	-1.68	-0.12	-1.85	-1.89	0.04
Ir	-1.22	-1.18	-0.04	-1.26	-1.32	0.06	-1.31	-1.25	-0.06	-1.20	-1.34	0.14
Pt	-1.27	-1.58	0.31	-1.20	-1.35	0.15	-1.40	-1.35	-0.05	-1.22	-1.32	0.10
Hf	-2.08	-1.52	-0.56	-2.12	-1.30	-0.82	-2.20	-1.32	-0.87	-2.08	-1.29	-0.79
Ta	-1.86	-1.34	-0.52	-1.81	-1.21	-0.60	-1.81	-1.25	-0.57	-2.31	-1.30	-1.01
W	-2.30	-1.67	-0.63	-2.20	-1.58	-0.62	-1.91	-1.55	-0.36	-1.78	-1.57	-0.20
Re	-2.07	-1.75	-0.32	-1.91	-1.37	-0.54	-2.08	-1.62	-0.46	-2.02	-1.37	-0.65
Cu	-0.93	-1.72	0.79	-0.48	-1.48	1.00	-0.99	-1.38	0.39	-0.61	-1.46	0.85
Ag	-0.71	-0.33	-0.38	-0.36	-0.50	0.14	-0.59	-0.57	-0.03	0.19	0.03	0.16
Au	-0.55	-1.12	0.58	-0.23	-0.96	0.73	-0.42	-0.86	0.44	-0.12	-0.71	0.59

Table S5. The MOs energies of *α, β*-UALs (E_{HOMO} and E_{HOMO-1}, eV) and their difference (E_{HOMO}-$E_{(HOMO-1)}$, eV).

	E_{HOMO}	E_{HOMO-1}	E_{HOMO}-$E_{(HOMO-1)}$
C_3H_4O	-0.49	-2.00	1.51
C_4H_6O	-0.41	-1.64	1.23
C_5H_8O	-0.46	-1.48	1.02
2-pentenal	-0.54	-1.75	1.21
Isophorone	-0.49	-1.62	1.13

Table S6. Electron number in *d*-orbital (θ_d) of the central metal atoms, Pauling electronegativity (E_M) and the first ionization energy (*IE*) of all used elements in CE.

Atom	θ_d	E_M	*IE*
Sc	1	1.36	631
Ti	2	1.54	658
V	3	1.63	650
Cr	5	1.66	653
Mn	5	1.55	717
Fe	6	1.83	759
Co	7	1.88	758
Ni	8	1.92	737
Cu	10	1.90	746
Y	1	1.22	616
Zr	2	1.33	660
Nb	4	1.59	664
Mo	5	2.16	685
Tc	6	1.91	702
Ru	7	2.20	711
Rh	8	2.28	720
Pd	10	2.20	805
Ag	10	1.93	731
Hf	2	1.32	654
Ta	3	1.51	761
W	4	2.36	770
Re	5	1.93	760
Os	6	2.18	840
Ir	7	2.20	880
Pt	9	2.28	870
Au	10	2.54	890
C	-	2.55	1086
N	-	3.04	1402

Table S7. The descriptor (λ) and the corresponding λ' for different models.

Atom	λ (C_3H_4O-M_1@C_2N)	λ (C_3H_4O-M_1@*g*-C_3N_4)	λ (C_3H_4O-M_1@DG)	λ (C_3H_4O-M_1@*h*-BN)	λ (C_4H_6O-M_1@C_2N)	λ (C_4H_6O-M_1@*g*-C_3N_4)	λ (C_4H_6O-M_1@DG)	λ (C_4H_6O-M_1@*h*-BN)
Sc	1.117	1.089	0.774	0.681	0.909	0.886	0.63	0.554
Ti	2.336	2.28	1.617	1.421	1.901	1.855	1.316	1.156
V	3.458	3.38	2.398	2.257	2.814	2.751	1.951	1.837
Cr	5.189	5.638	4.015	3.787	4.222	4.587	3.267	3.081
Mn	6.338	6.868	4.405	4.146	5.157	5.589	3.585	3.374
Y	0.981	1.061	0.756	0.566	0.798	0.863	0.615	0.461
Zr	2.454	2.279	1.621	1.211	1.996	1.854	1.319	0.985
Nb	4.719	4.591	3.265	2.967	3.84	3.735	2.657	2.414
Mo	6.078	5.944	4.215	3.964	4.945	4.836	3.43	3.225
Tc	7.472	7.311	5.185	4.87	6.08	5.949	4.219	3.962
Fe	8.077	7.867	5.6	6.013	6.572	6.401	4.557	4.892
Co	9.396	9.167	6.529	7.427	7.645	7.459	5.313	6.043
Ni	10.453	11.314	7.256	8.266	8.506	9.206	5.904	6.726
Ru	8.83	8.588	6.13	5.757	7.184	6.988	4.988	4.684
Rh	10.22	9.948	7.089	6.659	8.316	8.094	5.768	5.418
Pd	14.27	13.886	9.894	9.329	11.611	11.299	8.051	7.591
Os	8.722	8.753	6.207	5.823	7.097	7.122	5.05	4.738
Ir	10.39	11.795	7.579	7.111	8.454	9.597	6.167	5.786
Pt	13.543	13.499	9.628	9.066	11.02	10.984	7.834	7.377
Hf	2.323	2.259	1.606	1.304	1.89	1.838	1.307	1.061
Ta	4.049	3.949	2.806	2.641	3.295	3.213	2.283	2.149
W	5.467	5.348	3.79	3.561	4.448	4.352	3.084	2.897
Re	6.745	6.599	4.677	4.392	5.488	5.37	3.805	3.574
Cu	13.222	12.872	9.181	8.665	10.758	10.474	7.47	7.05
Ag	11.602	12.588	8.982	7.92	9.44	10.242	7.308	6.444
Au	16.126	15.328	10.942	9.658	13.121	12.472	8.903	7.858

Atom	λ (C_5H_8O-M_1@C_2N)	λ (C_5H_8O-M_1@*g*-C_3N_4)	λ (C_5H_8O-M_1@DG)	λ (C_5H_8O-M_1@*h*-BN)	λ (2-pentenal-M_1@C_2N)	λ (2-pentenal-M_1@*g*-C_3N_4)	λ (2-pentenal-M_1@DG)	λ (2-pentenal-M_1@*h*-BN)
Sc	0.749	0.73	0.519	0.457	0.898	0.875	0.622	0.547
Ti	1.566	1.529	1.084	0.953	1.877	1.832	1.300	1.142
V	2.319	2.266	1.608	1.513	2.779	2.716	1.927	1.814
Cr	3.479	3.78	2.692	2.539	4.170	4.530	3.227	3.043
Mn	4.25	4.605	2.954	2.78	5.093	5.519	3.540	3.332
Y	0.658	0.711	0.507	0.38	0.788	0.853	0.607	0.455
Zr	1.645	1.528	1.087	0.812	1.972	1.831	1.302	0.973
Nb	3.164	3.078	2.189	1.989	3.792	3.689	2.624	2.384

Mo	4.075	3.985	2.826	2.658	4.884	4.776	3.387	3.185
Tc	5.01	4.902	3.476	3.265	6.004	5.875	4.166	3.913
Fe	5.416	5.275	3.755	4.031	6.491	6.322	4.500	4.832
Co	6.3	6.146	4.378	4.979	7.550	7.366	5.247	5.968
Ni	7.009	7.585	4.865	5.542	8.400	9.091	5.831	6.642
Ru	5.92	5.758	4.11	3.86	7.095	6.901	4.926	4.626
Rh	6.852	6.67	4.753	4.465	8.212	7.994	5.697	5.351
Pd	9.568	9.31	6.634	6.255	11.467	11.158	7.951	7.496
Os	5.848	5.869	4.162	3.904	7.009	7.034	4.988	4.679
Ir	6.966	7.908	5.081	4.768	8.349	9.478	6.090	5.714
Pt	9.081	9.051	6.455	6.078	10.883	10.848	7.736	7.285
Hf	1.557	1.515	1.077	0.874	1.867	1.815	1.291	1.048
Ta	2.715	2.647	1.881	1.77	3.254	3.173	2.254	2.122
W	3.665	3.586	2.541	2.387	4.393	4.298	3.046	2.861
Re	4.522	4.425	3.136	2.945	5.420	5.303	3.758	3.529
Cu	8.865	8.63	6.156	5.81	10.624	10.343	7.378	6.963
Ag	7.779	8.44	6.022	5.31	9.323	10.115	7.217	6.364
Au	10.812	10.277	7.336	6.475	12.958	12.317	8.792	7.761

Atom	λ' (M_1@C_2N)	λ' (M_1@*g*-C_3N_4)	λ' (M_1@DG)	λ' (M_1@*h*-BN)
Sc	0.740	0.721	0.513	0.451
Ti	1.547	1.510	1.071	0.941
V	2.290	2.238	1.588	1.495
Cr	3.436	3.734	2.659	2.508
Mn	4.197	4.548	2.917	2.746
Y	0.650	0.703	0.501	0.375
Zr	1.625	1.509	1.074	0.802
Nb	3.125	3.040	2.162	1.965
Mo	4.025	3.936	2.791	2.625
Tc	4.948	4.842	3.434	3.225
Fe	5.349	5.210	3.709	3.982
Co	6.223	6.071	4.324	4.919
Ni	6.923	7.493	4.805	5.474
Ru	5.848	5.687	4.060	3.813
Rh	6.768	6.588	4.695	4.410
Pd	9.450	9.196	6.552	6.178
Os	5.776	5.797	4.111	3.856
Ir	6.881	7.811	5.019	4.709
Pt	8.969	8.940	6.376	6.004
Hf	1.538	1.496	1.064	0.864
Ta	2.681	2.615	1.858	1.749
W	3.621	3.542	2.510	2.358
Re	4.467	4.370	3.097	2.909
Cu	8.756	8.525	6.080	5.738
Ag	7.683	8.336	5.948	5.245
Au	10.679	10.151	7.246	6.396

Table S8. Calculated MOs energies of *α, β*-UALs (E_{HOMO} and $E_{(HOMO-1)}$, eV) and their difference (E_{HOMO}-$E_{(HOMO-1)}$, eV) by B3LYP/6-31g.

	E_{HOMO}	E_{HOMO-1}	E_{HOMO}-$E_{(HOMO-1)}$
C_3H_4O	-7.05	-8.00	0.95
C_4H_6O	-6.80	-7.51	0.71
C_5H_8O	-6.64	-7.10	0.46
2-pentenal	-6.72	-7.35	0.63

Table S9. The predicted adsorption energies (predicted ΔE_{ads}, eV) with the DFT-calculated results (DFT ΔE_{ads}, eV) for C_3H_4O adsorbed on M_1-doped C_2N, the predicted ΔE_{ads} with the DFT ΔE_{ads} for isophorone adsorbed on M_1@C_2N, and the descriptor λ with the DFT ΔE_{ads} of isophorone adsorbed on M_1@*h*-BN (B_v).

Atom	C_3H_4O on M_1-doped C_2N		isophorone on M_1@C_2N		isophorone on M_1@*h*-BN (B_v)	
	predicted ΔE_{ads}	DFT ΔE_{ads}	predicted ΔE_{ads}	DFT ΔE_{ads}	λ	DFT ΔE_{ads}
Sc	-0.65	-0.74	-0.77	-1.05	0.543	-0.88
Ti	-0.53	-0.49	-0.65	-0.46	1.366	-0.83
V	-0.42	-0.26	-0.53	-0.60	2.025	-0.46
Cr	-0.19	-0.25	-0.35	-0.52	3.393	-0.34
Mn	-0.13	-0.10	-0.23	-0.59	3.723	-0.25
Y	-0.65	-0.67	-0.78	-1.00	0.530	-0.83
Zr	-0.53	-0.55	-0.63	-0.43	1.135	-0.73
Nb	-0.30	-0.23	-0.40	-0.43	2.285	-1.06
Mo	-0.16	-0.13	-0.26	-0.06	2.9500	-0.60
Tc	-0.03	0.26	-0.12	-0.07	3.628	-0.27
Fe	0.08	0.05	-0.05	-0.18	4.729	-0.19
Co	0.19	0.26	0.08	-0.09	5.522	-0.05
Ni	0.32	0.32	0.19	0.08	6.131	0.02
Ru	0.11	0.32	0.03	0.44	4.29	-0.59
Rh	0.24	0.43	0.17	0.18	5.48	-0.18
Pd	0.64	0.31	0.59	0.57	6.935	0.07
Os	0.12	0.18	0.01	0.06	4.342	-0.32
Ir	0.37	0.32	0.19	0.14	6.400	0.19
Pt	0.60	0.38	0.51	0.39	6.741	0.36
Hf	-0.53	-0.77	-0.65	-0.58	1.125	-1.12
Ta	-0.36	-0.31	-0.47	-0.58	1.963	-1.03
W	-0.22	-0.21	-0.32	-0.36	2.653	-0.69
Re	-0.10	0.18	-0.19	-0.20	3.272	-0.47
